# Telomere length and micronuclei trajectories in APP/PS1 mouse model of Alzheimer's disease: Correlating with cognitive impairment and brain amyloidosis in a sexually dimorphic manner

**DOI:** 10.1111/acel.14121

**Published:** 2024-03-07

**Authors:** Xihan Guo, Jianfei Li, Yanmei Qi, Juanlin Chen, Minyan Jiang, Lina Zhu, Zetong Liu, Han Wang, Gongwu Wang, Xu Wang

**Affiliations:** ^1^ School of Life Sciences, The Engineering Research Center of Sustainable Development and Utilization of Biomass Energy Yunnan Normal University Kunming Yunnan China; ^2^ Yeda Institute of Gene and Cell Therapy Taizhou Zhejiang China

**Keywords:** Alzheimer's disease, amyloid β, genome instability, micronuclei, Morris water maze test, sexual dimorphism, telomere length

## Abstract

Although studies have demonstrated that genome instability is accumulated in patients with Alzheimer's disease (AD), the specific types of genome instability linked to AD pathogenesis remain poorly understood. Here, we report the first characterization of the age‐ and sex‐related trajectories of telomere length (TL) and micronuclei in APP/PS1 mice model and wild‐type (WT) controls (C57BL/6). TL was measured in brain (prefrontal cortex, cerebellum, pituitary gland, and hippocampus), colon and skin, and MN was measured in bone marrow in 6‐ to 14‐month‐old mice. Variation in TL was attributable to tissue type, age, genotype and, to a lesser extent, sex. Compared to WT, APP/PS1 had a significantly shorter baseline TL across all examined tissues. TL was inversely associated with age in both genotypes and TL shortening was accelerated in brain of APP/PS1. Age‐related increase of micronuclei was observed in both genotypes but was accelerated in APP/PS1. We integrated TL and micronuclei data with data on cognition performance and brain amyloidosis. TL and micronuclei were linearly correlated with cognition performance or Aβ_40_ and Aβ_42_ levels in both genotypes but to a greater extent in APP/PS1. These associations in APP/PS1 mice were dominantly driven by females. Together, our findings provide foundational knowledge to infer the TL and micronuclei trajectories in APP/PS1 mice during disease progression, and strongly support that TL attrition and micronucleation are tightly associated with AD pathogenesis in a female‐biased manner.

## Graphical abstract

Our study provides foundational knowledge to infer the telomere length and micronuclei trajectories in APP/PS1 mice during disease progression, strongly supports that telomere length attrition and micronucleation are tightly associated with AD pathogenesis in a female‐biased manner.

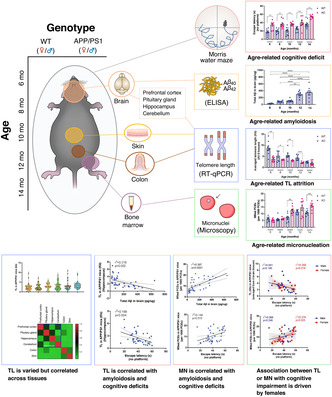

**Abbreviations**
AD: Alzheimer disease
ANOVA: analysis of variance
APP: amyloid precursor protein
Aβ: amyloid β
BCA: bicinchoninic acid
cGAS: cyclic guanosine monophosphate‐adenosine monophosphate (cGAMP) synthase
DSBs: double‐strand breaks
ELISA: Enzyme‐linked immunosorbent assay
GIN: genome instability
MN: micronuclei
MNed: micronucleated
PCEs: polychromatic erythrocytes
PS1: presenilin‐1
RT‐qPCR: reverse transcription quantitative polymerase chain reaction
STING: stimulator of interferon genes
TL: telomere length
WT: wildtype

## 1 INTRODUCTION

Alzheimer's disease (AD), the most common cause of dementia in the world, is characterized by the progressive cognitive impairment, loss of memory and language skill. AD presents two major diagnostic hallmarks: the accumulation of extracellular amyloid‐β (Aβ) plaques and intracellular phosphorylated Tau. These two hallmarks are strongly associated with the initiation and progression of AD. However, targeting these two proteins has largely failed to demonstrate the clinical efficacy (Karran & De Strooper, 2022). Thus, this field should be expanded to examine what we have less known, such as dissecting the active role of genome instability (GIN) in AD.

Generally, cell genome is stressed by exogenous and endogenous insults. These insults induce different classes of somatic mutations, ranging from single‐nucleotide variants, insertion–deletions, copy‐number variants in some genomic regions to large structural variants, and whole‐chromosome aneuploidy. Single genome sequencing has revealed that neurons, despite without cell division, accumulate somatic mutations at a constant rate throughout life (Abascal et al., 2021). There is an increased DNA double‐strand break accumulation and reduced DNA repair function in the hippocampus of AD brains compared to the non‐AD control brains (Thadathil et al., 2021). A recent study sequenced the genome of 319 neurons from AD cases and health controls and found that single‐nucleotide variants are increased in individuals of AD (Miller et al., 2022). More recently, genome structural variations and 3D genome disorganization induced by persistent DNA double‐strand breaks (DSBs) are critical pathological steps in the progression of AD (Dileep et al., 2023). A dramatic increase of chromosome 21‐specific aneuploidy has been detected in the cerebral cortex (Iourov et al., 2009) and buccal cells of AD (Thomas & Fenech, 2008). Chromosome X aneuploidy was also observed in brain cells of AD patients (Yurov et al., 2014). Despite these findings, our understanding of GIN in AD is still superficial. Telomere length (TL) alteration and chromosomes trapped within micronuclei (MN) are two GIN events well studied in cancer (Guo, Ni, et al., 2019); however, their occurrence and significance in AD are poorly explored.

Telomeres locate at the ends of eukaryotic linear chromosome and are made of tandem repeats of DNA and a set of associated proteins (Rossiello et al., 2022). Since chromosomes fail to completely replicate their 5′ end of each DNA strand, telomeric DNA is shortened with each round of cell division. If TL becomes too short, chromosomes become unstable due to DNA recombination and end‐joining events, which ultimately lead to cellular apoptosis or senescence. Multiple lines of evidence have shown that the TL of blood cells or brain cells from patients with AD is significantly shorter as compared with that in control subjects (Hochstrasser et al., 2012) and TL correlates with cognitive function (Canela et al., 2007) and with AD status (Boccardi et al., 2015). Recently, data from European‐ancestry participants in the UK Biobank (*n* = 435,046) reveals that longer TL is inversely associated with AD, cognitive impairment, and brain structural lesions toward the development of AD (Liu et al., 2023). The Mendelian randomization study has provided evidence for a causal role of TL shortening in AD (Scheller Madrid et al., 2020; Zhan et al., 2015). However, these results are mixed, as some studies reveal that TL is not significantly associated with AD condition (Hinterberger et al., 2017; Lukens et al., 2009), shorter TL is associated with better cognitive performance in AD patients (Liu et al., 2016), and longer TL is associated with increased risks of AD (Fani et al., 2020), especially in individuals of Aβ‐ and tau‐positive (Mahoney et al., 2019).

MN are small nucleus‐like bodies separated from the primary nucleus and usually resulted from the missegregated chromosomal fragments or entire chromosomes (Guo, Ni, et al., 2019). MN are not only a reliable biomarker of GIN, but they also have much severe consequences to the micronucleated chromosome and/or the host cells. Recent evidence supported that MN are also a strong mutator phenotype that can drive genetic heterogeneity through catastrophic events such as chromothripsis, a novel form of GIN in cancer cells, in the micronucleated chromosome (Guo, Dai, et al., 2021). Besides, the presence of MN acts as a trigger for cellular senescence and apoptosis. For example, MN with disrupted micronuclear envelope (Guo et al., 2020) promotes inflammatory signaling by introducing double‐stranded DNA into the cytosol, thereby engaging the cGAS‐STING antiviral pathway, which is a driving force of cellular senescence and neurodegenerative diseases (Guo et al., 2022). There is a significantly increased incidence of spontaneous MN in lymphocytes of AD patients than healthy controls (Petrozzi et al., 2002).

In this study, by assuming that TL and MN are more affected in AD, we profiled cognitive performance, Aβ, TL, and MN, in both sexes of APP/PS1 and the age‐matched wildtype (WT) mice. Our study is a major step forward in dissecting the TL and MN trajectories in AD mouse model and provides a unique opportunity for better understanding the role of TL attrition and micronucleation in AD pathogenesis.

## 2 MATERIALS AND METHODS

### 2.1 Mice

In our study, we used the previously described APP/PS1 mice (C57BL/6 background) overexpressing amyloid precursor protein (APP) and presenilin‐1 (PS1). APP/PS1 double transgenic mice were kindly provided by Dr. Keming Zhu and Hongliang Li (Yunnan University). APP/PS1 mice (Jax stock # 005864) was obtained by co‐injecting two plasmids encoding mutant human genes found in familial AD, one is the *APP* ^*swe*^ mutant and the other is the ΔE9 mutant of *PS1* (Jankowsky et al., 2001). Our choice of APP/PS1 was based on the facts that they develop progressive cognitive deficits and recapitulates several pathological hallmarks of AD, such as Aβ plaques, gliosis, synaptic degeneration, and neuronal loss since 6 months (Götz et al., 2018; Savonenko et al., 2005).

APP/PS1 mice were genotyped as described previously (Bai et al., 2019). Briefly, offspring were tail snipped in the second postnatal week and DNA was extracted from tail tissue as previously described (Yusof et al., 2019). The genotypes of each offspring were determined by polymerase chain reaction (PCR), using primers specific for the *APP* and *PS1* sequence of the APP/PS1 construct (forward GACTGACCACTCGACCAGGTTCTG; AATAGAGAACGGCAGGAGCA; reverse: CTTGTAAGTTGGATTCTCATATCCG; GCCATGAGGGCACTAATCAT). The β‐actin was used as an internal reference using the specific primers (forward: GCTACAGCTTCACCACCACAG; reverse: CGTCTTTACGGATGTCAACGTC). Offspring mice not expressing APP/PS1 transgenic construct were regarded as WT littermates, which were used as age‐matched controls.

Mice were housed in same‐sex groups of 4–6 per cage at the Yunnan Normal University under standard conditions at a temperature of 22°C and a 12 h light/dark cycle with ad libitum access to standard maintenance diet (Keao‐Xieli Feed Co., Ltd, Beijing, China) and water. Eighty mice aged 6–14 months comprising both sexes and both APP/PS1 and WT were randomly grouped, and four mice were included in each age group per genotype. Animal care, handing, genotyping, and experiments were approved by Yunnan Normal University biomedical research ethical committees and were in accordance with the Regulations for the Administration of Laboratory Animals (Yunnan province, China).

### 2.2 Morris water maze test

As previously described (Wang & Wang, 2016), we used a 180‐cm‐diameter tub containing a circular transparent plastic platform (12 cm in diameter) submerged 1.5 cm below the water surface in the Morris water maze test. The tub was filled with water to a depth of about 30 cm. Water was maintained at 22 ± 1°C and colored with white paint (titanium dioxide) to prevent animals from seeing the underwater platform. The tub was surrounded by a curtain ornamented with several marked visual cues. A video‐tracking system above the tub was used to record the swimming track of the animal. Behavioral data were recorded and analyzed by a computer installed with a visual analyzing system (Xeye™ ABA; MacroAmbition S&T Development Co. Ltd., Beijing, China).

The whole water surface was equally divided into four quadrants (southeast, southwest, northeast and northwest) without physical boundaries. Twenty‐four hours prior to the formal experiments, mice were allowed to swim freely for 120 s to adapt to the tank environment. In the following 6 days (days 1–6), each mice underwent four trials (60 s for each trial, interval = 5 min) per day. During each round trial, mice were released from each of four compass points (southeast, southwest, northeast, and northwest) in a randomized order. If a mouse did not find the hidden platform within 60 s, it was gently guided by researchers to the platform and remained on the platform for 20 s. If a mouse could find the platform within 60 s, it was allowed to stay on the platform for 10 s. The escape latency of each trial was recorded. All mice were towel dried and returned to their home cage at the end of each trial.

After 6 days of training, mice were subjected to a single 60‐s probe trial that took place 24 h after the last training trial (day 7). The platform was removed, and mice were placed on the opposite wall at the point furthest from the former platform location. During the probe trial, the latency of the first time to the platform region, the total times of target platform crossing, and the relative time spent in the target quadrants were recorded to evaluate spatial memory of platform location. All behavioral tests were performed during animal's light cycle between 1 PM and 6 PM in a sound attenuated environment.

### 2.3 Tissue preparation

All mice were killed by mechanical cervical dislocation. The brain was removed and bisected into right and left hemispheres. Then, the prefrontal cortex, pituitary gland, hippocampus, and cerebellum were isolated carefully from the right hemispheric brains. The left hemispheres were collected for Aβ analyses. In addition, a piece of colon (0.5 cm in length) was dissected from each mouse. After being shaved with an electric shaver, a piss of abdomen skin (0.5 cm^2^ in area) was dissected from each mouse and the fat and underlying subcutis were removed with a scalpel. All these dissected tissues were snap‐frozen in liquid nitrogen and stored at −80°C until they were used for RT‐qPCR and ELISA analysis. Meanwhile, bone marrow for MN analysis was collected by perfusing through the femurs with ice‐cold saline.

### 2.4 Absolute TL assessment by RT‐qPCR

Frozen tissues (prefrontal cortex, pituitary gland, hippocampus, cerebellum, colon, and skin) were cut into pieces of approximately 1 mm^2^ with a scissor and mechanically homogenized with a sonic homogenizer in 2 mL of PBS. Each sample was sonicated continuously until all pellets dissolved. The homogenized cells were collected after centrifugation at 12,000 *g* for 5 min. DNA was extracted according to the manufacturer's instructions (Tiangen, China). TL assessment was carried by RT‐qPCR, as previously described (Hu et al., 2020; Wang et al., 2018). The RT‐qPCR reactions were performed on the StepOne Plus instrument (Applied Biosystems, Foster City, CA, USA).

Briefly, a telomere standard constituted by an 84 bp DNA oligonucleotide (14 repeats of TTAGGG) and an oligonucleotide standard for mouse 36B4 (The acidic ribosomal phosphoprotein PO gene), a single‐copy gene used to determine the genome copy number, were synthesized. These two oligonucleotide standards were serially diluted with double‐distilled water to produce five concentrations. Using these DNA samples, standard curves were generated for TL and for 36B4 copy amplification reactions in StepOne Plus. Meanwhile, for analysis of sample DNA, triplicate PCR reactions using 20 ng of each DNA were carried out in a 20 μL volume using the Power SYBR Green Master mix (Roche). As previously described (O'Callaghan & Fenech, 2011), the primer sequences for telomere and 36B4 are 36B4‐F: ACTGGTCTAGGACCCGAGAAG, 36B4‐R: TCAATGGTGCCTCTGGAGATT. Telo‐1: CGGTTTGTTTGGGTTTGGGTTTGGGTTTGGGTTTGGGTT, Telo‐R: GGCTTGCCTTACCCTTACCCTTACCCTTACCCTTACCCT. The cycling condition for each primer was initially denatured at 94°C for 10 min, followed by 40 cycles of 94°C for 5 s (denaturation), 58°C for 15 s (annealing), and 72°C for 40 s (extension). The qPCR data was processed and analyzed as previously described (O'Callaghan & Fenech, 2011), which gave a total TL in kb per mice diploid genome.

### 2.5 Measurement of Aβ

Dissected left hemispheric brain of each mouse were homogenized with eight volumes of RIPA buffer containing protease inhibitor, followed by 2‐h incubation on ice. Supernatants were collected after 30 min of centrifugation at 12,000 *g*. Bicinchoninic acid (BCA) protein assay was used to measure the protein concentrations of each sample. Commercial mice Aβ ELISA kits (Meibiao, Jiangsu, China) were used to quantify Aβ_40_ and Aβ_42_ performed with supernatants at room temperature as per the manufacturer's instructions. Concentrations were calculated from measurement of absorbance read at 450 nm with SpectraMax iD3 (Molecular Devices). Finally, the levels of Aβ_40_ and Aβ_42_ were normalized to total protein concentration measured by BCA protein assay.

### 2.6 Bone marrow MN assay

The bone marrow MN assay was conducted as previously described (Guo, Qi, et al., 2021; Guo, Su, et al., 2023). Briefly, after mouse was killed by cervical dislocation, both femurs of each mouse were dissected. Then, the femurs were flushed with 3 mL ice‐cold saline supplemented with 10% newborn calf serum (Sigma). The cell suspension was transferred into a centrifuge tube and was centrifuged at 1000 rpm for 5 min. After the supernatant was removed, the pellet was fully resuspended in 0.5–1.5 mL of saline (with 10% newborn calf serum). Then, cells were prepared onto glass slides by cytospin centrifugation and fixed by ice‐cold methanol at −40°C for 15 min. After this, slides were air‐dried and stained with 10% Giemsa (in PBS, pH 6.8). For each mouse, at least three slides were prepared and each slide was randomly assigned to a trained research staff member to perform cell counts under an optical microscope with 1000× magnification (Olympus, Tokyo, Japan). At least 4000 polychromatic erythrocytes (PCEs) were screened per mouse for the presence of MN and the number of micronucleated (MNed) PCEs per 1000 PCEs in each mouse was calculated.

### 2.7 Statistical analysis

Statistical analysis was performed using GraphPad Prism version 7 for Windows (GraphPad Software, USA). Data were first tested for normality using the Shapiro–Wilk test and all the datasets were normally distributed. Unpaired Student's *t* test was used to estimate the difference between two groups. One‐way and two‐way analysis of variance (ANOVA) with Tukey's post hoc tests were used to estimate the difference among three or more groups. The significance of the correlation between two datasets was measured by the linear regression analyses. Pearson's correlation coefficients (*r* ^2^) were calculated to indicate the strength of the relationship between two variables. The slope of the regression line was used to calculate the rate of TL shortening. Data were presented as mean ± SEM. A *p* < 0.05 was considered significant (*p < 0.05, **p < 0.01, ***p < 0.001, and ****p < 0.0001), and those >0.05 were considered nonsignificant. The exact analysis method was indicated in figure legends and the text.

## 3 RESULTS

### 3.1 APP/PS1 mice exhibit progressively accelerated cognition decline versus age‐matched WT mice

**FIGURE 1:**
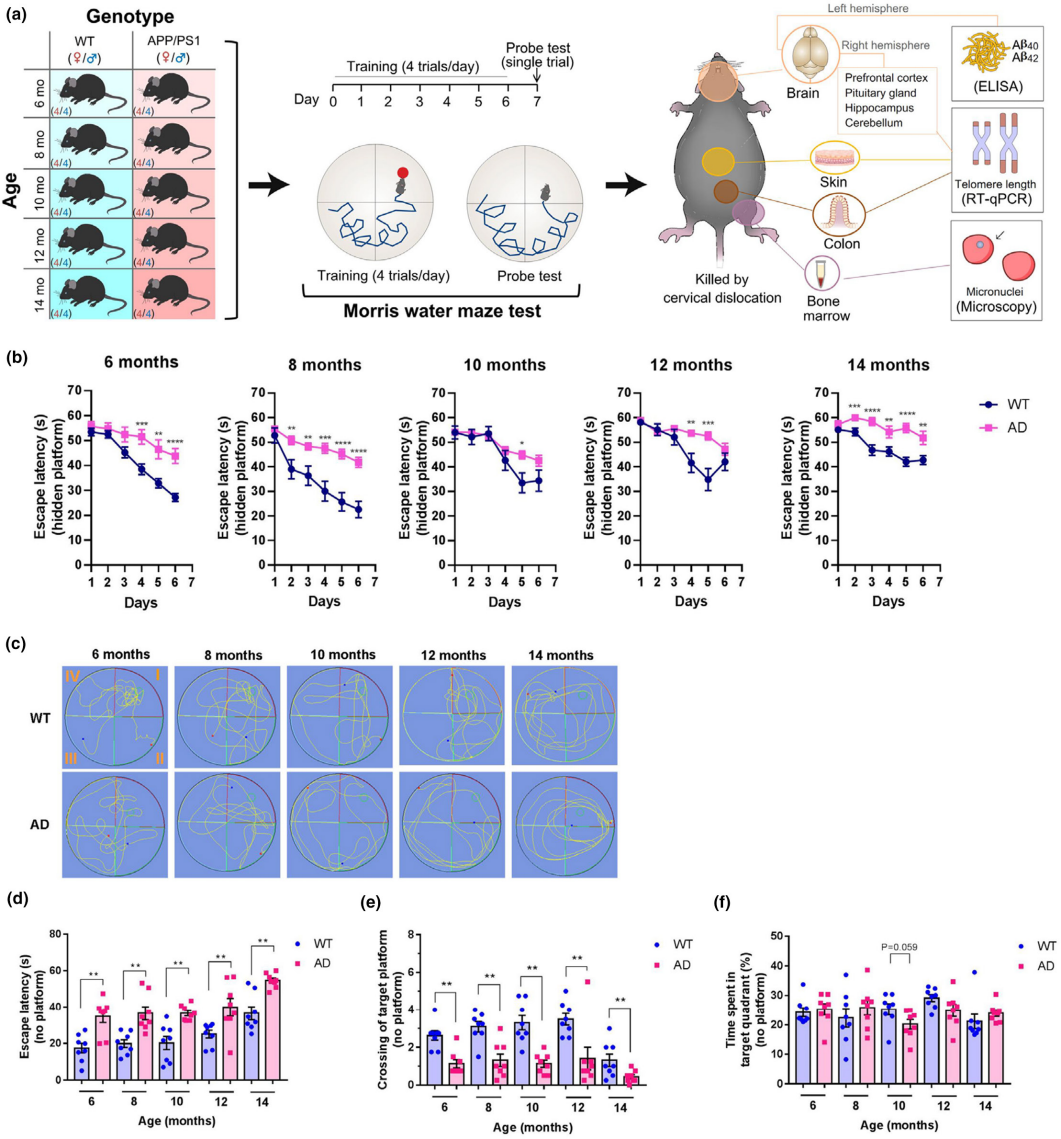
Age‐dependent changes of cognitive performances in APP/PS1 and WT mice. (a) The overview of workflow in this study. (b–f) The results of Morris water maze test. (b) The time to find the hidden platform (escape latency) during the six training days in 6‐, 8‐, 10‐, 12‐, 14‐month‐old APP/PS1 and WT mice. (c) Representative images of the swimming tracks of mice at day 7 (probe test) in the Morris water maze test. (d) First time to the platform region during the probe test (escape latency). (e) Platform crossing times in a probe test. (f) Relative time spent in the target quadrant. All values in (b) and (d–f) are shown as mean ± SEM. Statistical analysis used in (b) and (d–f) is a two‐tailed Student's *t* test. *p < 0.05, **p < 0.01, ***p < 0.001, and ****p < 0.0001. Each dot represents an individual sample.

We initially established a group of mice consisting of 20 male and 20 female APP/PS1 mice between 6 and 14 months of age. These time points represent critical stages in AD phenotypes development and progression in APP/PS1 mice (Jankowsky et al., 2001). In parallel, we also established a group of mice consisted of 20 male and 20 female WT mice between 6 and 14 months of age (Figure 1a). The weight between APP/PS1 and WT mice from each age group was not significantly different at age 6–12 months. In 14‐month‐old mice, there was a significant increase in bodyweight for the APP/PS1 compared to WT (34.50 ± 0.67 g, APP/PS1; 29.46 ± 1.21 g, WT; *p* < 0.01).

At 6, 8, 10, 12, and 14 months of age, we assessed the cognition ability of APP/PS1 and/or WT mice via the Morris water maze test (Figure 1a). We found that, compared to the age‐matched WT mice, APP/PS1 mice display worsened spatial learning and memory in their age from 6 to 14 months (Figure 1b–f). In the training trials, the time to find the hidden platform (escape latency) progressively decreased from days 1 to 6 at all the measured age points; however, APP/PS1 mice took significantly longer to find the platform than WT mice in their age from 6 to 14 months (Figure 1b). Compared with the WT mice, the AD model mice showed obvious memory deficits in the post‐training probe trial on day 7, as evidenced by more random motion paths (Figure 1c), increased time to find the platform region for the first time (Figure 1c; *F* = 7.056 and *p* < 0.001 in WT mice, *F* = 6.274 and *p* < 0.001 in APP/PS1 mice), and decreased times of target platform crossing at day 7 (Figure 1e; *F* = 7.818 and *p* < 0.001 in WT mice, *F* = 1.281 and *p* = 0.296 in APP/PS1 mice). Among the markers analyzed for cognition performance, relative time spent in the target quadrant (Figure 1f) was not significantly different between age‐matched APP/PS1 and WT mice in every age groups.

Taken together, our data demonstrated that APP/PS1 mice showed impaired spatial learning and reduced memory retention, as compared with the age‐matched WT mice, at each age group. The obvious cognitive deficit in APP/PS1 mice was evident as early as 6 months of age.

### 3.2 APP/PS1 mice have an age‐related increase of brain Aβ load that correlates with cognition performance

**FIGURE 2:**
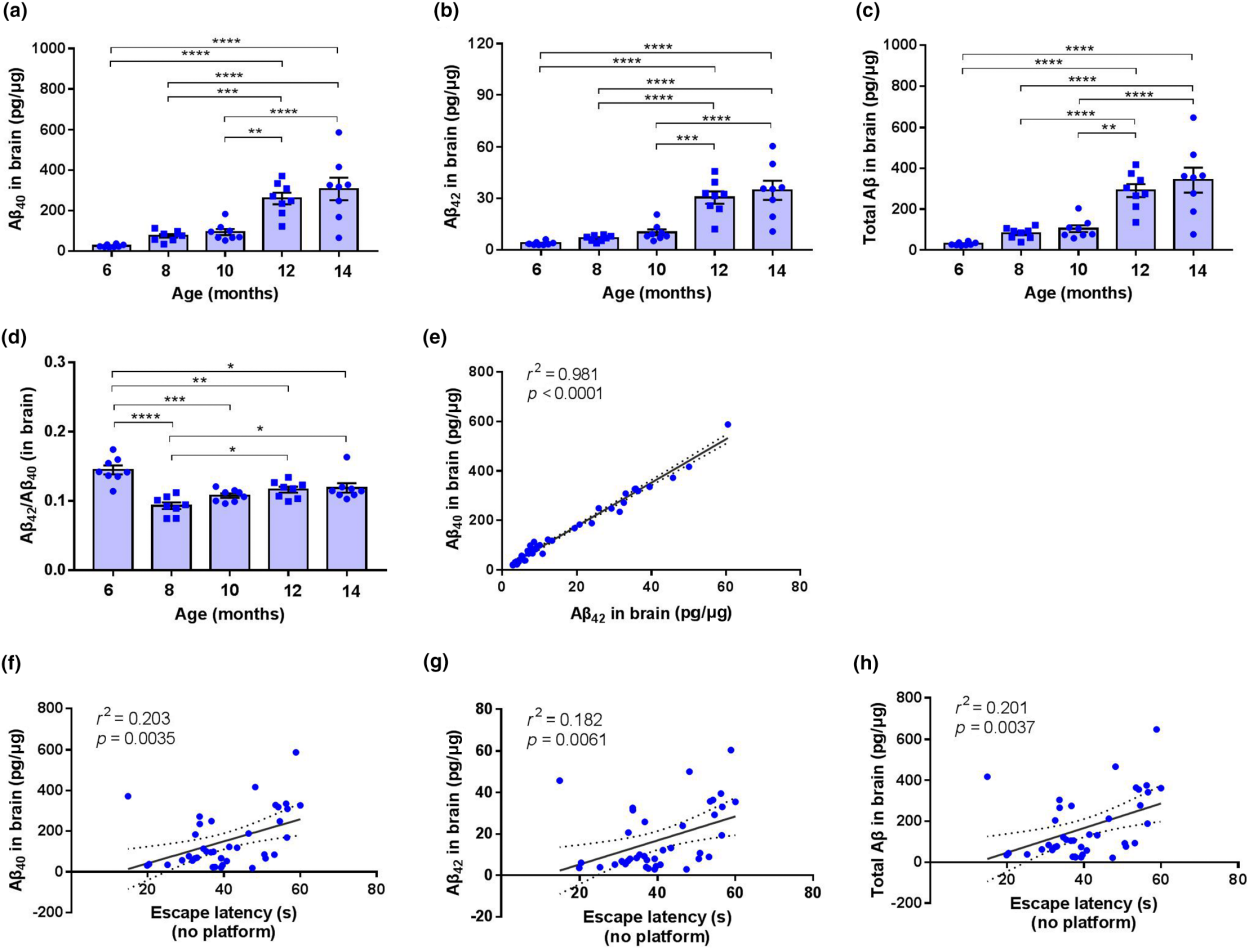
Aβ is increased in APP/PS1 mice and correlated with cognitive performance. (a–c) The quantifications of brain Aβ_40_, Aβ_42_, and the total Aβ in APP/PS1 mice at ages 6–14 months (*n* = 8 in each age group). (d) The ratios of Aβ_42_/Aβ_40_ based on Aβ_42_ and Aβ_40_ concentrations in APP/PS1 mice brain. (a–d) **p < 0.01, ***p < 0.001, ****p < 0.0001, two‐sided one‐way ANOVA followed by Tukey's posttest. (e) The correlation between Aβ_40_ and Aβ_42_. (f–h) Correlation between escape latency and Aβ_40_, Aβ_42_, or the total Aβ. (e–h) Linear regression (*n* = 40 unless otherwise stated). *r* ^2^ and *p* values from linear regression are represented in each panel.

Following the behavioral tests, mice were killed and the brain Aβ loads in APP/PS1 mice at the ages of 6–14 months were assessed by ELISA (Figure 1a). Aβ mainly consists of 40 or 42 amino acids (Aβ_40_ and Aβ_42_, respectively). The level of Aβ_40_ (Figure 2a), Aβ_42_ (Figure 2b), and total Aβ (Figure 2c) significantly increased with age (*F* = 18.13 and *p* < 0.0001 for Aβ_40_, *F* = 21.55 and *p* < 0.0001 in for Aβ_42_, *F* = 18.5 and *p* < 0.0001 in for total Aβ). At 6 months of age, all APP/PS1 mice had low levels of Aβ_40_ (26.45 ± 2.41 pg/μg) and Aβ_42_ (3.82 ± 0.38 pg/μg). The concentrations of both Aβ species were suddenly elevated at the age of 12 months (260.70 ± 28.48 and 30.57 ± 3.61 pg/μg for Aβ_40_ and Aβ_42_, respectively). By the age of 14 months, the concentrations of Aβ_40_ and Aβ_42_ increased by 11.6‐folds (307.85 ± 55.53 pg/μg) and 9.1‐folds (34.726 ± 5.59 pg/μg), respectively, as compared to 6 months. In addition, there is a substantial variation in Aβ_40_ and Aβ_42_ concentrations in APP/PS1 among different individual mouse at the age of 14 months (Figure 2a,b).

Since Aβ_42_/Aβ_40_ ratio has implicated in familial AD and has been suggested to be a better indicator of AD pathology than total Aβ (Ashton et al., 2022), we calculated the ratio of brain Aβ_42_/Aβ_40_ of each mouse. The data showed that age‐related changes in the ratio of Aβ_42_/Aβ_40_ were not linear (*F* = 13.08 and *p* < 0.001) as maximal index was observed at 6 months of age (Figure 2d). At 8 months of age, APP/PS1 mice showed a dramatically decreased ratio of Aβ_42_/Aβ_40_. Beyond 8 months of age, the ratio of Aβ_42_/Aβ_40_ tended to increase progressively. These data suggested that, in APP/PS1 mice, brain Aβ_42_/Aβ_40_ ratio is not a major driver for cognitive deficits. Interestingly, we found that the levels of Aβ_40_ and Aβ_42_ were proportional in all APP/PS1 mice. Pearson correlation analyses revealed a near‐perfect correlation between Aβ_40_ and Aβ_42_ (*r* ^2^ = 0.981, *p* < 0.0001; Figure 2e), suggesting that most Aβ_40_ and Aβ_42_ were produced simultaneously in APP/PS1 mice.

When cognitive data from 6‐ to 14‐month‐old mice were pooled together and correlated with Aβ_40_ and Aβ_42_ load, significant correlations were revealed for the concentration of Aβ_40_ (*r* ^2^ = 0.203, *p* = 0.0035; Figure 2f), Aβ_42_ (*r* ^2^ = 0.182, *p* = 0.0061; Figure 2g), and total Aβ (summed from Aβ_40_ and Aβ_42_, *r* ^2^ = 0.201, *p* = 0.0037; Figure 2h) with escape latency. Positive values of these correlations indicated that a higher load of Aβ_40_, Aβ_42_, or total Aβ coincided with a poorer performance in the learning task, and vice versa. Unexpectedly, no significant correlation was demonstrated between the ratio of Aβ_42_/Aβ_40_ and escape latency (Figure S1A), even only 8‐ to 14‐month‐old mice were included (Figure S1B). In addition, no significant correlation was demonstrated between the Aβ_40_ or Aβ_42_ concentration with times of target platform crossing (Figure S2).

Thus, our results agree with the previous finding that there is a strong correlation between memory deficits and total Aβ loads, but not the Aβ_42_/Aβ_40_ ratio, in the brain of APP/PS1 mice (Savonenko et al., 2005), suggesting that the Aβ load is a reliable and robust predictor of cognitive deficits in APP/PS1 mice.

### 3.3 APP/PS1 mice have an overall shortened TL across tissues versus age‐matched WT mice

Next, we attempted to determine the absolute TL in AD and WT mice at 6–14 months of age using the RT‐qPCR (Figure 1a). To better understand the variation in TL, we measured absolute TL in six different tissue types: prefrontal cortex, cerebellum, pituitary gland, hippocampus, colon, or skin.

**FIGURE 3:**
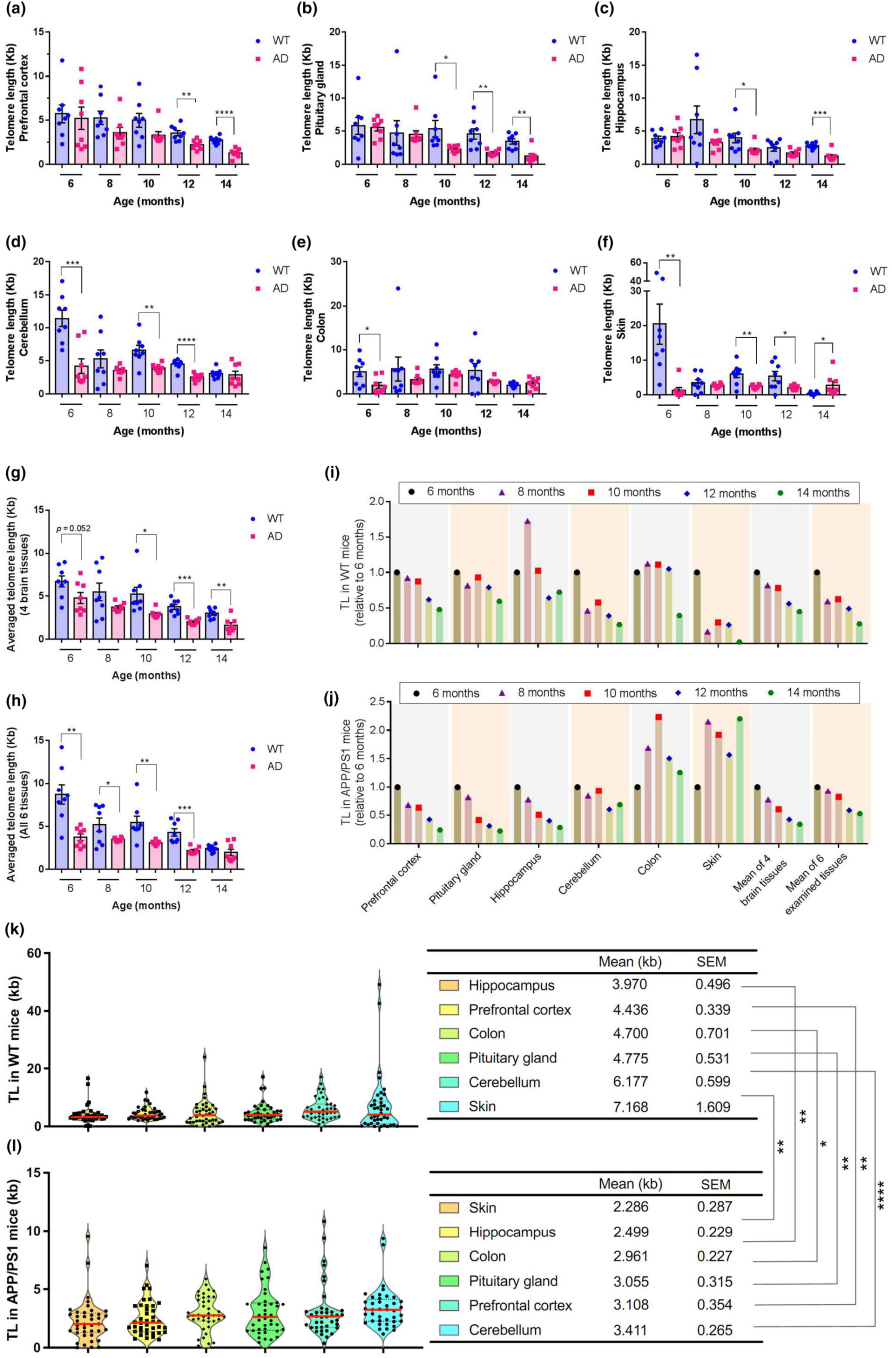
Age‐dependent shortening of telomere length (TL) in different tissues of APP/PS1 and WT mice. (a–f) Distribution of TL in prefrontal cortex (a), pituitary gland (b), hippocampus (c), cerebellum (d), colon (e), and skin in APP/PS1 and WT mice of indicated age. (g, h) The mean TL from four brain tissue types or from all six examined tissues. (i, j) Age‐related changes of TL across tissues relative to 6‐month mice. (k, l) TL differs across tissue types in WT and APP/PS1 mice. Violin plots show the distribution of TL (ordered by mean TL) in WT (a) and APP/PS1 (b) mice at age from 6 to 14 months ranked by mean TL across all measured tissue types. Data are shown as both a single point originating from a single individual and a smoothed violin plot to represent the population distribution. The horizontal line in (a) and (b) represent the median. We found no significant difference in the mean TL across different tissue types in either WT or APP/PS1 mice (two‐sided one‐way ANOVA followed by Tukey posttest). The mean TL in matched tissues was significant longer in WT mice than APP/PS1 mice (two‐tailed Student's *t* test). All values in (a–h) are shown as mean ± SEM. Statistical analysis used in (a–h), (k) and (l) is two‐tailed Student's *t* test. *p < 0.05, **p < 0.01, ***p < 0.001, and ****p < 0.0001.

We first compared tissue‐specific TL between AD and WT mice. The results showed TL across tissues in APP/PS1 mice tended to be shorter than age‐matched WT mice (Figure 3a–f). In prefrontal cortex, TL of APP/PS1 mice became significantly shorter than that of WT mice at the 12 months of age (Figure 3a). In pituitary gland (Figure 3b) and hippocampus (Figure 3c), TL of APP/PS1 mice became significantly shorter than of WT mice at the 10 months of age. In cerebellum (Figure 3d) and skin (Figure 3f), TL of APP/PS1 mice became significantly shorter than of WT mice at the 6 months of age. In colon, significantly shortened TL in APP/PS1 mice was only observed at 6 months of age (Figure 3e). To further compare the difference of TL between AD and WT mice, we analyzed the averaged TL from four brain tissues or all six examined tissues. The results showed that there was a significant difference in brain TL between age‐matched AD and WT mice at 10 months of age and beyond (Figure 3g). TL averaged from six tissues was significantly shorter in APP/PS1 mice than this in WT mice at all tested ages except 14 months of age (Figure 3h).

Next, we analyzed age‐related change of TL across different tissues in WT and APP/PS1. We calculated the age‐related changes in TL relative to 6 months of age in each tissue at different time points. The data revealed that in all six tissues, there was an age‐associated decrease in TL in WT mice (Figure 3i), providing evidence that age‐related TL shortening occurs in most tissue types. Age‐related TL attrition in APP/PS1 mice was only evidenced in four brain tissues (Figure 3j). In APP/PS1 mice, TL of colon and skin did not correlate with age at ages from 6 to 14 months. For example, colon TL in 10‐month APP/PS1 mice was twofold longer than this of 6‐month mice (Figure 3j). Similar result was also observed in skin TL of APP/PS1 mice, as we observed the TL of 14‐month mice was twofold longer than 6‐month mice (Figure 3j).

When we compared the TL of matched tissues between WT and APP/PS1 mice, three interesting phenomena were observed (Figure 3i,j). First, the TL of four specific brain regions were consistently decreased in an age‐related manner in both genotypes, suggesting TL attrition is a common phenomenon during natural aging and AD progression. Second, colon TL was largely homeostatic by the age from 6 to 14 months in both genotypes, indicating TL homeostasis is essential to ensure high turnover rate of colon. Third, skin TL was decreased in WT mice but increased in APP/PS1 mice in age‐dependent ways. We speculate the cause of these inconsistent results is probably due to high interindividual variation in TL in mice population. Since our study was not longitudinal, the TL data in mice at any of the studied age points did not reflect the TL dynamics in a given individual over time.

In addition, we compared the mean TL of WT and APP/PS1 mice with different ages. On average, TL was the shortest in hippocampus and longest in skin in WT mice (Figure 3k). We estimated the contribution of age and tissue type to the variation in TL using two‐way ANOVA models. Age and tissue type explained 28.57% (*p* = 0.0004) and 4.42% (*p* = 0.0281) of the variation in TL across all tissues, respectively, indicating that age accounts for substantial variability in WT mice TL. On average, TL was the shortest in skin and longest in cerebellum in APP/PS1 mice (Figure 3l). In APP/PS1 mice, age and tissue type explained 28.75% (*p* = 0.0003) and 4.42% (*p* = 0.0276) of the variation in TL, respectively, indicating that age accounts for substantial variability in APP/PS1 mice TL. To further explore differences in TL by genotype, we compared mean TL of same tissues from WT and APP/PS1 mice. In each tissue type, the mean TL was significantly higher in WT as compared with APP/PS1 (Figure 3k,l), with the largest difference in skin (3.14‐fold higher) and smallest in prefrontal cortex (1.43‐fold higher).

**FIGURE 4:**
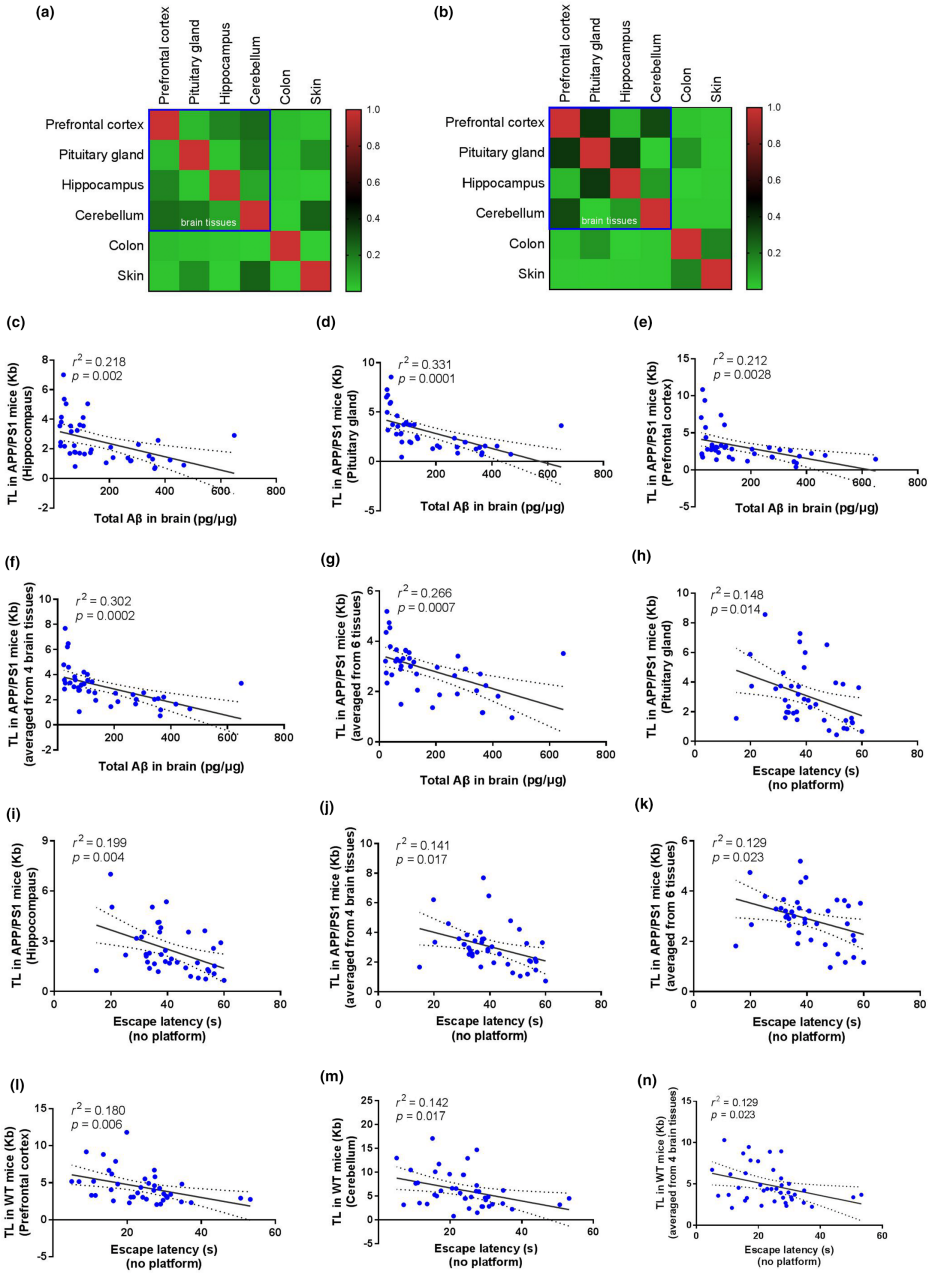
Correlations between telomere length (TL) with Aβ or cognition performance. (a, b) Pearson correlations (*r* ^2^) between TL measures from different tissue type in WT (a) and APP/PS1 mice (b). (c–k) Correlations between TL with Aβ and cognition performance in APP/PS1 mice. (c–e) Scatterplots of the total Aβ for TL in hippocampus (c), TL in pituitary gland (d), and TL in prefrontal cortex (e). (f, g) Scatterplots of the total Aβ for the mean TL from four brain tissue types (f), and the mean TL from six examined tissues (g). (h–k) Scatterplots of the escape latency for TL in pituitary gland (h), TL in hippocampus (i), the mean TL from four brain tissue types (j), and the mean TL from six examined tissues (k). (l–n) Correlation between TL and cognition performance in WT mice. Scatterplots of the escape latency for TL in prefrontal cortex (k), TL in cerebellum (l), and the mean TL from four brain tissue types (m). Linear regression analysis was used (*n* = 40 unless otherwise stated). The black line denotes the line of best fit and the shaded area between two dashed lines represents the 95% confidence interval. *r* ^2^ and *p* values from linear regression are represented in each panel.

Although TL varied across different tissue types in a given individual in both genotypes, TL across different brain regions were generally positively correlated, especially in APP/PS1 mice (Figure 4a,b, Figures S3 and S4). In APP/PS1 mice, pituitary gland TL was a proxy for TL in other tissues, because it had the strongest correlation with average TL measurement from six tissues (*r* ^2^ = 0.595, *p* < 0.0001; Figure S5). In WT mice, skin TL was a proxy for TL in other tissues, because it had the strongest correlation (*r* ^2^ = 0.669, *p* < 0.0001) with average TL measurement from six tissues (Figure S6).

Together, these data demonstrate that (i) there is a substantial variation in TL in either WT or APP/PS1 mice; (ii) TL variation in both genotypes is attributable to tissue type and age; and (iii) APP/PS1 mice have a shorter TL in brain tissues than WT mice, suggesting important differences in telomere maintenance in APP/PS1 compared to WT depending on sampled tissue.

### 3.4 Aβ level and/or cognition deficit correlates with TL to a greater extent in APP/PS1 compared to WT

To identify TL changes in APP/PS1 and WT mice that may be linked to AD pathology, we conducted linear regression analysis in both APP/PS1 and WT mice. In three out of four brain tissue types, TL was negatively correlated with total Aβ concentration of brain, with Pearson correlations (*r* ^2^) ranging from 0.212 to 0.331 (Figure 4c–e; Figure S7A). Moreover, in these brain tissues, TL was negatively correlated with both Aβ_40_ and Aβ_42_ (Figure S7B–I). Either the TL of colon or skin was correlated with total Aβ concentration of brain (Figure S7J,K). To fortify the link between TL and Aβ level, we calculated the mean TL from four brain tissues or from six examined tissues. The mean TL from four brain tissues remained a high Pearson correlation (*r* ^2^ = 0.302) with total Aβ concentration (Figure 4f), but was attenuated (*r* ^2^ = 0.266) if the mean TL was calculated from all six examined tissues (Figure 4g).

In two out of four brain tissue types, TL was negatively correlated with escape latency in Morris water maze test, with Pearson correlations (*r* ^2^) ranging from 0.148 to 0.199 (Figure 4h,i; Figure S8A,B). Interestingly, hippocampus TL had the highest Pearson correlation to escape latency among the four brain tissue types (*r* ^2^ = 0.199; Figure 4i), indicating that TL attrition in hippocampus is highly linked to AD progression. Either the TL of colon or skin was correlated with escape latency (Figure S8C,D). The mean TL from four brain tissues remained a high Pearson correlation (*r* ^2^ = 0.141) with escape latency (Figure 4j). The Pearson correlation was attenuated (*r* ^2^ = 0.129) if the mean TL was calculated from all six examined tissues (Figure 4k).

Upon finding the significant association between TL and Aβ level and/or cognition performance in APP/PS1 mice, we wanted to explore whether such association also held in WT mice. However, we found that, in WT mice, the TL of prefrontal cortex and cerebellum (Figure 4l,m), but not pituitary gland, hippocampus, colon, or skin, was significantly associated with escape latency in Morris water maze test (Figure S9A–D). Moreover, the mean TL from four brain tissue types (Figure 4n), but not this from six examined tissues (Figure S9E), was significantly associated with escape latency.

Overall, our data supports that TL of brain tissues is negatively associated with Aβ load and cognitive deficits among APP/PS1 mice, but to a less extent among WT mice.

### 3.5 APP/PS1 mice have an elevated incidence of MN in bone marrow versus age‐matched WT mice

Next, we examined the landscape of MN frequency in bone marrow of WT and APP/PS1 mice at ages from 6 to 14 months (Figure 1a). Measuring the number of MN in mammalian red blood cells is relatively straightforward because PCEs (erythrocyte precursor cells that reside in the bone marrow) expel their nuclei during red blood cell development, leaving behind any genetic materials that would form into one or more MN (Guo, Su, et al., 2023).

**FIGURE 5:**
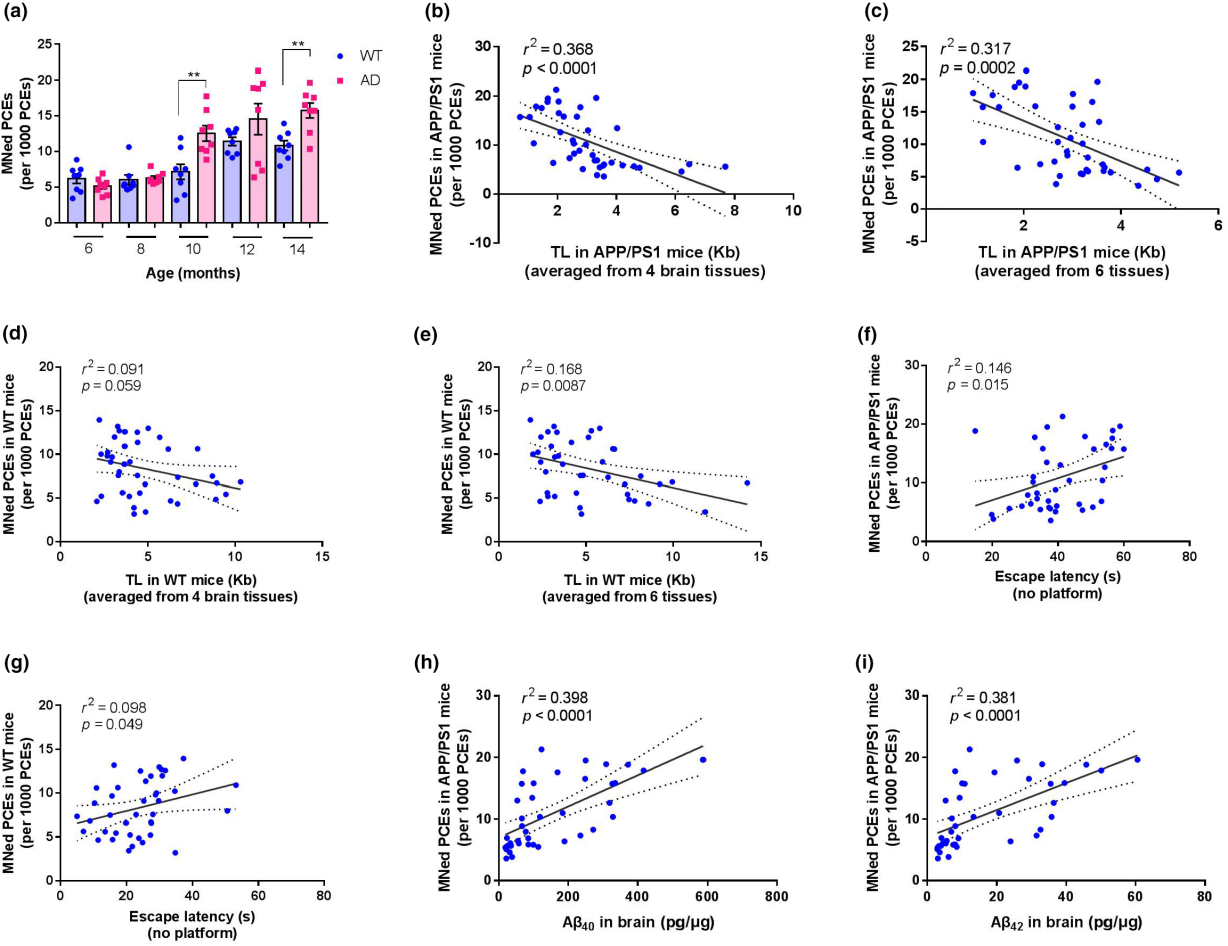
Age‐related increase of micronuclei (MN) in bone marrow and its correlation with telomere length (TL), cognition performance, and Aβ loads. (a) Distribution of MN in bone marrow in APP/PS1 and WT mice of indicated age. (b–e) Age‐related changes in TL and MN are correlated. Scatterplots of the MN frequency for the mean TL from four brain tissue types (b, d), and the mean TL from six examined tissues (c, e) in APP/PS1 mice (b, c) and WT mice (d, e). (f–i) Correlations between MN frequency and Aβ or cognition performance in APP/PS1 and WT mice. Scatterplots of the MN frequency in bone marrow for escape latency in APP/PS1 (a) and WT (b) mice. Scatterplots of the MN frequency in bone marrow for Aβ_40_ (c) and Aβ_42_ (d). All values in (a) are shown as mean ± SEM and two‐tailed Student's *t* test was used in (a). **p < 0.01. Linear regression analysis was used in (b–i) (*n* = 40 unless otherwise stated). The black line denotes the line of best fit and the shaded area between two dashed lines represents the 95% confidence interval. *r* ^2^ and *p* values from linear regression are represented in each panel.

The data showed that both the WT and APP/PS1 mice displayed an age‐dependent increase in the frequency of MN in PCEs (*F* = 11.9, *p* < 0.0001 in WT mice; *F* = 16.1, *p* < 0.0001 in APP/PS1 mice). In WT mice, the frequency of MN in 12‐ and 14‐month mice was significantly higher than this in 6‐, 8‐, and 10‐month mice (all *p* < 0.05, Figure 5a). In APP/PS1 mice, the frequency of MN in 10‐, 12‐, and 14‐month mice was significantly higher than this in 6‐ and 8‐month mice (all *p* < 0.01, Figure 5a). These data indicated that the age‐dependent increase of MN was accelerated in APP/PS1 mice. At 10–14 months of age, there was a main effect of genotype on the frequency of MN. At the ages of 10 and 14 months, the frequency of MN in APP/PS1 mice was significantly higher than this in WT mice (*p* < 0.01). A similar pattern was also observed by 12 months of age, though no significance was reached (Figure 5a).

We also examined the relationship between MN and TL in APP/PS1 and WT mice. The data showed that, in APP/PS1 mice, MN was negatively associated with the mean TL from four brain tissue types (Figure 5b) or from six examined tissues (Figure 5c). The strength of such association was slightly attenuated in WT mice. We observed no significant association between MN and mean TL from four brain tissue types (Figure 5d). Across all examined tissues, the mean TL was negatively correlated with MN in WT mice (Figure 5e).

These results demonstrate that age‐related increase of micronucleation is accelerated in APP/PS1 mice, and age‐related changes in TL and MN correlated significantly in both APP/PS1 and WT mice, albeit more pronounced in APP/PS1 mice, suggesting cells with short TL are more vulnerable to undergo micronucleation.

### 3.6 Cognition performance negatively correlates with MN frequency in APP/PS1 mice, but to a lesser extent in WT mice

To determine whether MN variation among APP/PS1 and WT mice had any functional significance, we examined the association between MN and cognition performance. The data showed that there was a strong positive correlation between MN and escape latency in APP/PS1 mice (Figure 5f) and a weak positive correlation between MN and escape latency in WT mice (Figure 5g). These data suggest that, in APP/PS1 mice, the correlation between MN and cognition performance was somewhat stronger compared with WT mice. The strong correlation observed in APP/PS1 mice led us to further examine whether MN was associated with Aβ. The results demonstrated that MN had a strong positive correlation with either Aβ_40_ (Figure 5h) or Aβ_42_ (Figure 5i).

### 3.7 Sex differences in TL and MN are observed in APP/PS1 and WT mice

Sex differences are extensively presented in the physiological systems of most animals including human. Since sex is a biological variable in preclinical research, both sexes of mice were included in our initial research designs. Here, we aimed to assess whether TL and/or MN differentially contributes to AD Pathology in both sexes, and therefore, we compared all the aforementioned major parameters between male and females in APP/PS1 and WT mice.

**FIGURE 6:**
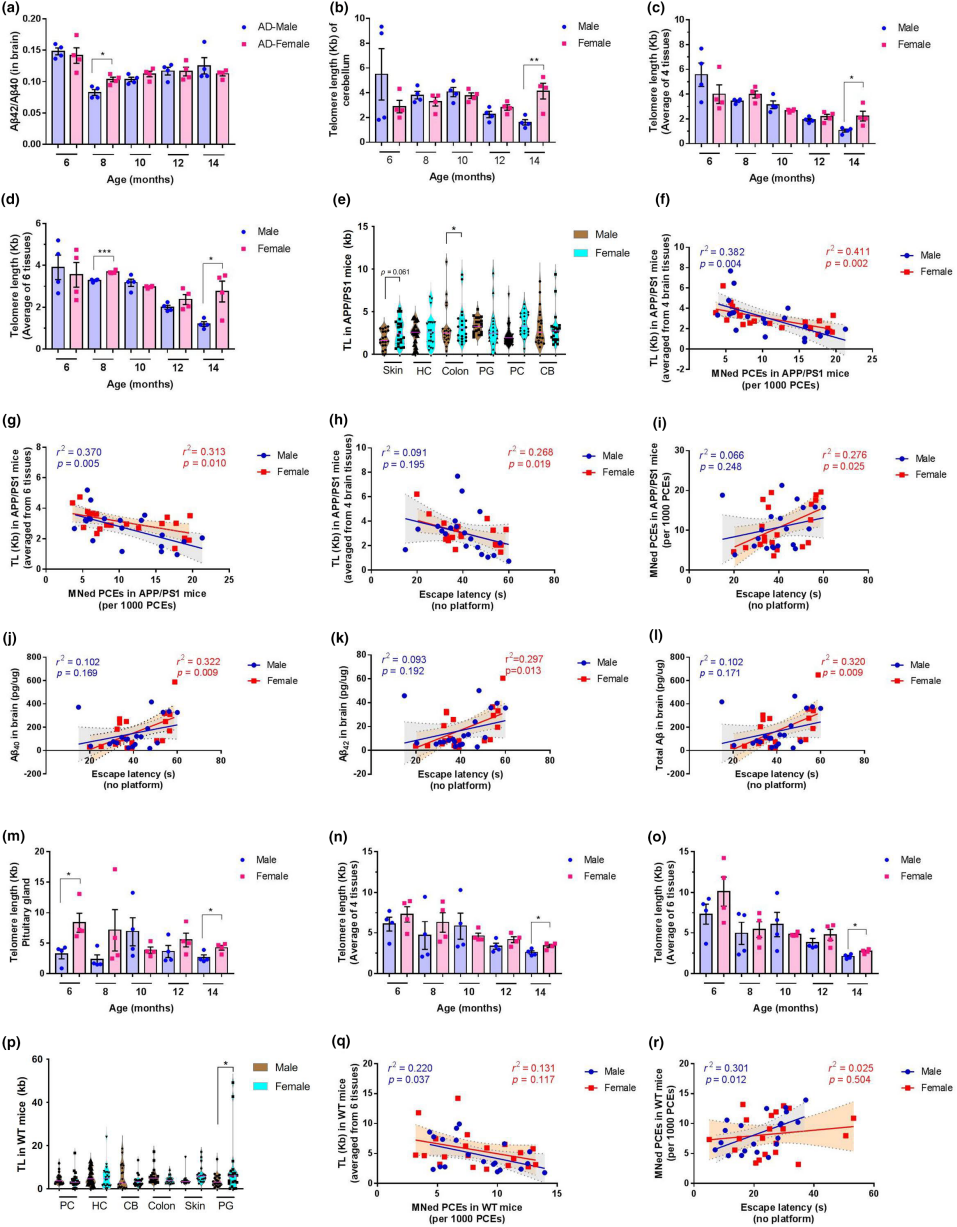
Sex‐dimorphic changes in APP/PS1 and WT mice. (a–l) Sex‐dimorphic changes in APP/PS1 mice. Age‐related changes in the ratio of Aβ_42_/Aβ_40_ (a), cerebellum TL (b), mean TL from four brain tissues (c) or six examined tissues (d) in male and female mice. (e) The mean TL across different tissues in male and female mice. Data are shown as both a single point originating from a single individual and a smoothed violin plot to represent the population distribution. (f, g) Sex‐specific association between mean TL from four brain tissues (f) or six examined tissues (g) with MN frequency. Sex‐specific association between escape latency with mean TL from four brain tissues (h), MN frequency (i), Aβ_40_ (j), Aβ_42_ (k), and the total Aβ (l). (m–r) Sex‐dimorphic changes in WT mice. Age‐related changes in the pituitary gland TL (m), mean TL from four brain tissues (n) or six examined tissues (o) in male and female mice. (p) The mean TL across different tissues in male and female mice. Data are shown as both a single point originating from a single individual and a smoothed violin plot to represent the population distribution. (q) Sex‐specific association between mean TL from six examined tissues with MN frequency. (r) Sex‐specific association between escape latency with MN frequency. All values in (a–d) and (m–o) are shown as mean ± SEM. Statistical analysis used in (a–e) and (m–o) is two‐tailed Student's *t* test. *p < 0.05, **p < 0.01, and ***p < 0.001. The horizontal lines in (e) and (p) represent the median. Linear regression analysis was used in (f–l), (q) and (r) (*n* = 40 unless otherwise stated). The red (female) or blue lines (male) denotes the line of best fit and the shaded area between two dashed lines represents the 95% confidence interval. *r* ^2^ and *p* values of each sex from linear regression are represented in each panel. CB, cerebellum; HC, hippocampus; PC, prefrontal cortex; PG, pituitary gland.

In APP/PS1 mice, there was no significant difference in the cognitive performance, including escape latency and crossing of target platform, between age‐matched females and males (Figure S10A,B). Of the four parameters related to Aβ, only the ratio of Aβ_42_/Aβ_40_ was significantly different between sexes, with slightly higher level in females at the age of 8 months (Figure 6a; Figure S10C–E). These data demonstrated that, in APP/PS1 mice, there was no robust pathological difference between females and males.

In five out of six tissue‐specific TL, no significant difference between two sexes was evidenced at any age group (Figure S10F–J). Significant sex difference of tissue‐specific TL was limited to cerebellum. By the age of 14 months, females had a longer cerebellum TL than males (Figure 6b). This difference also held in case of mean TL from four brain tissue types (Figure 6c). Regarding mean TL from six examined tissues, it was significantly longer in females at ages of 8 and 14 months (Figure 6d). We compared the mean TL of each tissue obtained from male and female APP/PS1 mice and found that the mean TL of colon in females was significantly longer than in males (*p* < 0.05; Figure 6e). The mean TL of skin in females was longer than males, although the difference did not reach significance (*p* = 0.061; Figure 6e). This data points out that sex difference in TL is not consistent across all tissue types. In addition, MN frequency was not significantly differed between two sexes at any age point (Figure S10K).

To investigate whether sex modifies the association between AD phenotypes and GIN biomarkers, we tested for associations of five representative AD biomarkers, escape latency, Aβ_40_, Aβ_42_, total Aβ_40_, and the ratio of Aβ_42_/Aβ_40_, with tissue‐specific TL, mean TL, or MN frequency. We did this in the full dataset and separately in each sex using multivariable linear and logistic regression. If an effect fulfilled the following two criteria: (i) associations significant in the full mice group; (ii) associations significant in only one sex, it was considered sex‐specific. We found both sexes exhibited significant association between the mean TL of four brain tissue types (Figure 6f) or the mean TL of six examined tissues (Figure 6g), with MN frequency. We found that the association between the mean TL of four brain tissue types (Figure 6h), but not the mean TL of six tissues (Figure S10L), with escape latency was female specific. Although MN was not significantly associated with escape latency in males, it showed a significant positive association with escape latency in females (Figure 6i). Female‐specific effects were seen in association between Aβ and escape latency: Aβ_40_, Aβ_42_, or the total Aβ was significantly associated with escape latency in females, but not males (Figure 6j–l). These findings revealed that, while the abovementioned associations were strong in the full group analysis, yet it seemed that most of them are driven by female effects only, suggesting that TL shortening or MN increase is more relevant to AD in females.

Sex difference was less obvious in WT mice. There was no significant difference in the cognitive performance, including escape latency and crossing of target platform, between age‐matched females and males (Figure S11A,B). Significant sex difference of tissue‐specific TL was limited to pituitary gland, with females had a longer TL at the ages of 6 and 14 months (Figure 6m). A rough sex difference in tissue‐specific TL was seen in colon (Figure S11C) and skin (Figure S11D). No sex difference was seen in the TL of prefrontal cortex, hippocampus, or cerebellum (Figure S11E–G). Regarding the mean TL from four brain tissue types or six examined tissues, females possessed a significantly longer TL at the age of 14 months (Figure 6n,o). We compared the mean TL of each tissue obtained from the male and female WT mice and found that the mean TL of pituitary gland in females was significantly longer than in males (Figure 6p). As what observed in APP/PS1 mice, MN frequency was not significantly differed between two sexes in any age group (Figure S11H).

In WT mice, we revealed that males, but not females, exhibited significant association between the mean TL of six tissues (Figure 6q) with MN frequency. Regarding the mean TL form four brain tissues, it was not significantly associated with MN frequency in both sexes, albeit more pronounced in males (Figure S11I). The associations between the mean TL from four brain tissue types or six examined tissues with escape latency was not sex‐specific (Figures S11J and S10K). Interestingly, although MN was not significantly associated with escape latency in females, it showed a significant positive association with escape latency in males (Figure 6r), suggesting such association was driven by male effects only.

Together, these data revealed that (i) in APP/PS1 and WT mice, females have a somewhat longer TL but this pattern is not consistent across all tissue types; (ii) although APP/PS1 mice do not exhibit robust pathology between two sexes, the association between TL or MN with AD pathology is largely driven by female effects; and (iii) association between TL or MN with cognition decline in WT mice is largely driven by male effects. In summary, males and females display different trajectories of TL and MN, both at baseline and in amyloid pathology. All these findings highlighted the complexity in the potential roles of TL attrition and MN in AD pathology.

## 4 DISCUSSION

AD is a complex multifactorial disease that involves loss of genome stability. Identifying which specific GIN events that precede or coincide with AD onset or progression will significantly contribute to fully understanding this disease. Many unknowns exist when studying the roles of telomere shortening and MN in AD pathophysiology. Although mice models cannot exactly replicate human AD, they are tools for understanding the relationship between GIN and AD. In this regard, an optimal genetic model for such study should reflect the conditions in human patients. To the best of our knowledge, this is the first study that (i) systematically investigates age‐related trajectories in AD pathology, TL and MN in both sexes of APP/PS1 and WT mice, and (ii) conducts linear regression analysis to relate TL and MN changes to the cognitive and biochemical changes in both genotypes. Despite sex differences are well characterized in human AD, it is unclear whether TL and MN and they relations with AD phenotypes are different between sexes. Our data reveal that, while TL and MN do not display extensive difference between two sexes, their association to amyloidosis and cognitive deficit was more robust in females for APP/PS1 mice but was more robust in males for WT mice.

### 4.1 Accelerated TL attrition and micronucleation are dominant features of APP/PS1 mice

In this study, we measured TL in prefrontal cortex, cerebellum, pituitary gland, hippocampus, colon, and skin. The reasons for the focus on these tissues were as follows: (i) Although hippocampus is a well‐known tissue highly relevant to AD pathogenesis, the degeneration of prefrontal cortex, cerebellum, and pituitary gland is also involved in neuropathology of AD (Bossers et al., 2010; Han et al., 2015; Jacobs et al., 2018). We wanted to determine whether the TL of these brain regions are intercorrelated and to what extent do their TL correlate to AD pathogenesis; (ii) colon is a tissue that has a high rate of turnover, and we asked whether this tissue has ability to maintain TL homeostasis; (iii) skin is the first protective barrier against the external environment; thus, the skin TL is thought to be particularly susceptible to accelerated shortening due to both the internal and external insults (Buckingham & Klingelhutz, 2011). Our results provide a view of the substantial variation in TL that exists across tissue types and among individual mouse.

It has been known that the TL of buccal mucosa and olfactory bulbs in APP/PS1 mice was significantly shorter than WT at 12 months (Thomas et al., 2009). One of the strengths of our study is that the TL and MN assessment of the APP/PS1 and WT mice were conducted at different age points (6–14 months). Previously, the TL of blood cells and hippocampus has been assessed in 3xTg mice, an AD model expressing APP^swe^/PS1^M146V^/Tau^P301L^, at the ages of 5, 9, and 13 months (Martínez‐González et al., 2020). They found an age‐related decrease of TL in both examined tissues. In line with this study, we found an age‐related decrease of TL in all six examined tissues (prefrontal cortex, pituitary gland, hippocampus, cerebellum, colon, and skin). TL difference between 3xTg and WT mice was less evident in the study of Martínez‐González et al. (2020) and the significance was only achieved at the age of 13 months. In contrast, we found that TL difference between APP/PS1 and WT mice is extensive and depended on tissue type and age. Coupling these data, we anticipate that TL shortens more quickly in AD mice models and TL is varied across different tissues and varied among different AD mice models.

We also surveyed the MN trajectories in bone marrow from both WT and APP/PS1 mice with different age frames, which is the other strength of this study. To the best of our knowledge, this study is the first to survey TL and MN trajectories simultaneously in the same mice groups. We noted that MN frequency is negatively associated with TL in both genotypes, suggesting telomere shortening may be interconnected with micronucleation. While we found an age‐related increase in MN frequency in bone marrow, there was a significant difference in the frequency of micronucleated cells between WT and APP/PS1 mice by the age of 10 and 14 months. This finding is in line with the study of Thomas et al. (2009), which demonstrated there was a 1.7‐fold increase in micronucleated buccal cells and 1.3‐fold increase in the micronucleated PCEs occurred in the APP/PS1 group compared to the WT group, despite no significance achieved either.

However, it is worth noting that Thomas et al. (2009) did not observe an age‐related increase (between 6 and 12 months) in MN frequency in oral buccal cells or PCEs in APP/PS1 or WT mice. What might account for the differences between the studies' results? One possibility is the diet composition. The diet used in Thomas and colleagues' study was AIN‐93G diet, and the diet used in our study was standard maintenance diet. While these two diet types are broadly similar in their overall content of carbohydrates (65.08% vs. 64.3%), they differed in the contents of proteins (23.07% vs. 20.3%), fats (11.8% vs. 15.8%), and other specific micronutrients. In supporting with this possibility, we have previously shown that restriction on micronutrients or calorie has a great influence on MN frequency (Guo et al., 2017; Guo, Dai, et al., 2019; Guo, Qi, et al., 2021; Guo, Su, et al., 2023; Hu et al., 2020; Li et al., 2022; Yan et al., 2020).

Of note here, although aneuploidy—alterations in the number of chromosomes—is not the primary research point of this study, significant increase of aneuploidy has been observed in different cells of patients with AD. For example, in an early study, increased aneuploidy was found in blood cells of patients with sporadic AD, familial AD, or even unaffected siblings of the AD patients (Ward et al., 1979). Since critically short TL is a trigger of aneuploidy (Pampalona et al., 2010) and micronucleation is a biological process able to cause aneuploidy (Guo, Ni, et al., 2019), it is attractive to determine the arising order of these three GIN events in AD mice models. Moreover, telomere attrition and micronucleation have been suggested in driving the age‐related somatic mosaicism in structural and numerical chromosome alterations (Dai & Guo, 2021); it is therefore interesting to survey the landscape of age‐related mosaic chromosomal alterations in APP/PS1 mice.

### 4.2 Correlated age‐related changes of TL and MN with AD pathogenesis in APP/PS1 mice

Many mutant mouse strains have been developed as indispensable tools to investigate AD in humans (Fisher & Bannerman, 2019). No single mouse model can recapitulate all aspects neurodegenerative phenotypes in human AD because species‐specific differences exist in every aspect of human and mouse. A suggested agenda for working with mouse models of neurodegeneration is the molecular phenotypes we are studying in mice should be accurately modeling the appropriate phenotypes in humans (Fisher & Bannerman, 2019). There is a paradox in TL and life span, that is, mice have longer TL but shorter life span compared to human (Vera et al., 2012). This paradox has questioned about the value of mouse models in telomere field.

Indeed, studying the role of telomere in AD in mice models has produced controversial results. An early study has shown that despite TL shortening resulted from telomerase deficiency impairs adult neurogenesis and neuron maintenance, it can reduce Aβ plaque pathology in APP23 mice (Rolyan et al., 2011). Increased expression of telomerase in the brain of aged C57BL/6 mice with extremely short telomere due to telomerase deficiency was sufficient to ameliorate several neurodegeneration phenotypes (Whittemore et al., 2019). Although these data argue in favor of critically short telomere being a determinant of AD, there is one major confounding factor that should be considered. Telomerase‐induced amelioration may involve mechanisms other than its classical TL extending function. For instance, engineered telomerase expression in AD mouse model ameliorates AD phenotypes via upregulating the gene networks governing synaptic signaling and learning processes, a mechanism unrelated to its catalytic activity (Shim et al., 2021). Using a new method to specifically decelerate TL shortening in the cortex and myocardial tissue can effectively improve cognitive performance in a mouse model of AD (APP/PS1) (Zhang et al., 2023), suggesting TL is indeed a determinant of AD. These results indicate that a suitable mice model that can faithfully recapitulate TL attrition seen in human AD should be established to discern the functional consequence of TL attrition to AD pathogenesis.

In this study, we not only found an age‐related decrease in TL of APP/PS1 mice, but also demonstrated changes in TL and AD pathogenesis were linearly correlated. We noted that the significant decline of averaged TL among four brain tissues can be observed as early as the age of 6 months in APP/SP1 mice. By 6 months of age, significant decline of cognition performance was also observed in APP/PS1 mice. Thus, our findings suggest that TL shortening may be a very early foundational event in AD. Based on the critical role of telomere in cell physiology (Rossiello et al., 2022), we anticipate that short TL may trigger an important early part of the pathway network that are essential for AD onset and progression. Further clarification of telomere‐related pathogenesis in AD is greatly needed.

Like TL, MN is also linearly associated with cognition performance in both WT and APP/PS1 mice. Moreover, MN is significantly associated with Aβ load. Although MN is affected by AD progression in the APP/PS1 mice model, the increase of MN is delayed in time relative to A increase and cognition decline. Thus, it is tempting to speculate that micronucleation could not be the cause of AD, but more likely is the result of AD progression. Despite this, it is interesting to uncover whether MN exert some active role in driving AD progression, for example, by activating the cGAS‐STING pathway (Guo et al., 2022), which has an emerging role in driving age‐related neuroinflammation and neurodegeneration (Gulen et al., 2023; Xie et al., 2023). In this study, the age‐related landscape of MN was only surveyed in bone marrow. Currently, it is unknown to what extent can MN in bone marrow be used as a proxy for MN in other tissues, especially in brain tissues that are primarily composed of postmitotic cells and tightly associated with the pathogenesis of AD.

### 4.3 The associations between TL and MN with AD pathogenesis differ between sexes

The pathogenesis of AD exhibits sexual dimorphism, with females being 1.7 times more frequently afflicted than man (Cui et al., 2023). The mechanistic basis for this bias has not been fully estimated. The available evidence suggests that the main mechanisms for this sexual dimorphism include sex chromosome, sex hormone, and hypothalamic–pituitary–adrenal axis effects (Cui et al., 2023). Understanding the physiological differences between males and females in manifesting AD sexual dimorphism in both human patients and AD mice is one of the major challenges in AD research. We reasoned that, if GIN has critical role in AD pathogenesis, sex difference in GIN (Fischer & Riddle, 2018) might contribute to sex differences in AD.

In this study, we found limited evidence for sex‐biased TL, with females have significant longer TL than males in certain specific tissues at specific age. These results are consistent with the finding in human that TL is less influenced by sex (Demanelis et al., 2020). In addition, we found that the MN frequency in females and males is not significant differed. Although our data did not reveal an obvious sex difference in TL and MN, we noted that, in females, not males, TL and MN display consistent correlation curves with cognition performance, suggesting that TL attrition and MN increase are more relevant to AD pathogenesis in males. Our finding is consistent to the human data that females are more influenced by AD and, extend such influences to TL and MN. Thus, it will be essential to further explore the underlying mechanism.

An unexpected finding from our study is that the association between MN and cognition performance is differently modified by sexes between two genotypes. Unlike APP/PS1 mice, correlation curve between MN and cognition performance in WT mice was only observed in males but not females. These data indicate that the potential role of MN in memory loss in AD was fundamentally distinct from natural aging. In addition, it is very interesting if we link these data to the facts that men have shorter life span but women are more vulnerable to AD.

### 4.4 Limitations and future challenges

It is important to note that our study possesses several limitations that should be focused in the future. First, the mouse model of APP/PS1 does not fully capture the pathogenesis of human AD. We chose the mouse model that is featured by amyloid aspect, but not tau aspect, of human AD in this study. Follow‐up studies using tau models or combinatorial transgenic models (Götz et al., 2018) are necessary to confirm the correlation between AD phenotypes and TL attrition or MN increase. In addition, the validity of transgenic mice overexpressing APP and PS1 has been debated because these mice would suffer from artificial phenotypes derived from gene overexpression (Saito et al., 2014). To determine whether the phenotypes of accelerated TL attrition and MN that we observed in APP/PS1 mice are artifacts, similar validation using a knock in model system (Saito et al., 2014) that is physiologically closer to AD patients is also necessary.

Second, while TL was assessed in several brain tissues, MN was just measured in bone marrow. Currently, protocols for MN assessment in solid tissue is not standardized (Morita et al., 2011). Although bone marrow is widely used, because it can be easily obtained, whether it is a proxy for many other tissue types for MN assessment is poorly understood. A recently developed tool, called MATLAB‐based program for quantifying MN, would provide opportunity to quantitatively and accurately analyze MN in brain tissues of AD mice models (Yano et al., 2021). Interestingly, a recent study developed a fluorescence probe that is able to specifically target MN (Ding et al., 2023). They demonstrated that there is an age‐related increase of MN in cerebral cortex (but not hippocampus) of APP/PS1 mice, and the increase of MN is positively correlated with the Aβ deposition in cerebral cortex.

Third, although RT‐qPCR is widely used to detect TL, the TL obtained from this method is an average of the TL from all chromosomes. It has known that the shortest TL, not the average TL, is the key to determine cell viability, chromosome stability, and even life span (Hemann et al., 2001; Vera et al., 2012). This implies that even if the average TL is relatively long, telomere dysfunction may occur in a fraction of cells with very short TL. Thus, it will be important to test whether the percentage of cells with short telomere is increased in AD and whether it associates with or even determine disease progression, using the automated high throughput methods like quantitative fluorescence in situ hybridization (Canela et al., 2007).

Fourth, the observed robust decrease of TL and increase of MN in APP/PS1 could also be the result of AD progression. Recently, we have shown that Aβ_40_ and Aβ_42_ induce MN in human neuron and microglia cell lines (Guo, Jiang, et al., 2023). Moreover, Aβ_42_ has previously been shown to induce telomere DNA damage, telomere uncapping, chromosome fusion, and telomere shortening in tumor cell lines (Qin et al., 2019). In this study, we are unable to resolve the precise role of Aβ in telomere attrition and micronucleation in APP/PS1 mice. Future studies that perform long‐term Aβ immunotherapy in APP/PS1 mice will be better positioned to answer this question.

Despite these potential limitations, we believe that this study provides foundational knowledge to infer the TL and micronuclei trajectories in APP/PS1 mice during disease progression, and strongly support that TL attrition and micronucleation are tightly associated with AD pathogenesis in a female‐biased manner. The data generated in this study will provide a valuable resource for improving our understanding of TL and MN in AD and aging.

## AUTHOR CONTRIBUTIONS

**Xihan Guo:** Conception, methodology, data validation, writing—original draft, writing—review and editing, funding acquisition, and supervision. **Jianfei Li:** Methodology, data curation, and validation. **Yanmei Qi:** Data curation. **Juanlin Chen:** Data curation. **Minyan Jiang:** Data curation. **Lina Zhu:** Data curation. **Zetong Liu:** Data curation. **Han Wang:** Methodology. **Gongwu Wang:** Methodology. **Xu Wang:** Conception, resource, funding acquisition, and supervision. All authors contributed to the revision and review of this article.

## FUNDING INFORMATION

This work is funded by the National Natural Science Foundation of China (nos. 31900410 and 32260148 to X. G. and 31860301 to X. W.), the Yunnan Fundamental Research Projects (nos. 202001AU070055 and 202101AT070112 to X. G.), and the Outstanding Young Academics Award of Yunnan Normal University (2019 to X. G.).

## CONFLICT OF INTEREST STATEMENT

There are no conflicts to declare.

## Supporting information


Figure S1.
Figure S2.
Figure S3.
Figure S4.
Figure S5.
Figure S6.
Figure S7.
Figure S8.
Figure S9.
Figure S10.
Figure S11.

## Data Availability

The data that support the findings of this study are available from the corresponding author upon reasonable request.
