# Supplementary material for: Telomere length and micronuclei trajectories in APP/PS1 mouse model of Alzheimer's disease: Correlating with cognitive impairment and brain amyloidosis in a sexually dimorphic manner
Source: Aging Cell. 2024 Mar 7;23(5):e14121. doi: 10.1111/acel.14121 (PMC11113262; doi:10.1111/acel.14121)
Supplement: Supplementary file 1 — Figure S1. Figure S2. Figure S3. Figure S4. Figure S5. Figure S6. Figure S7. Figure S8. Figure S9. Figure S10. Figure S11. [file ACEL-23-e14121-s001.doc]

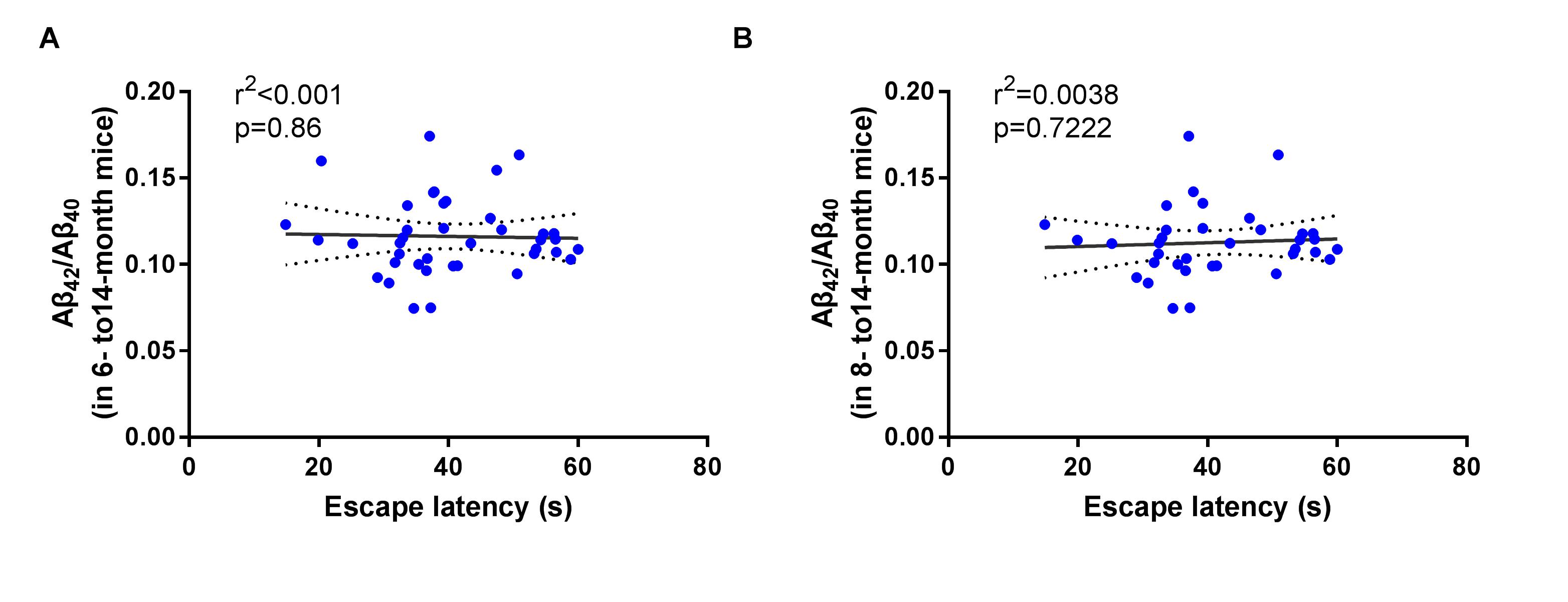


**Figure S1. Correlations between escape latency and the ratio of Aβ_42_/Aβ_40_ obtained from 6- to 14-month (A) or from 8- to 14-month (B) APP/PS1 mice.** Linear regression (n = 40 unless otherwise stated). r^2^ and p values from linear regression are represented in each panel.


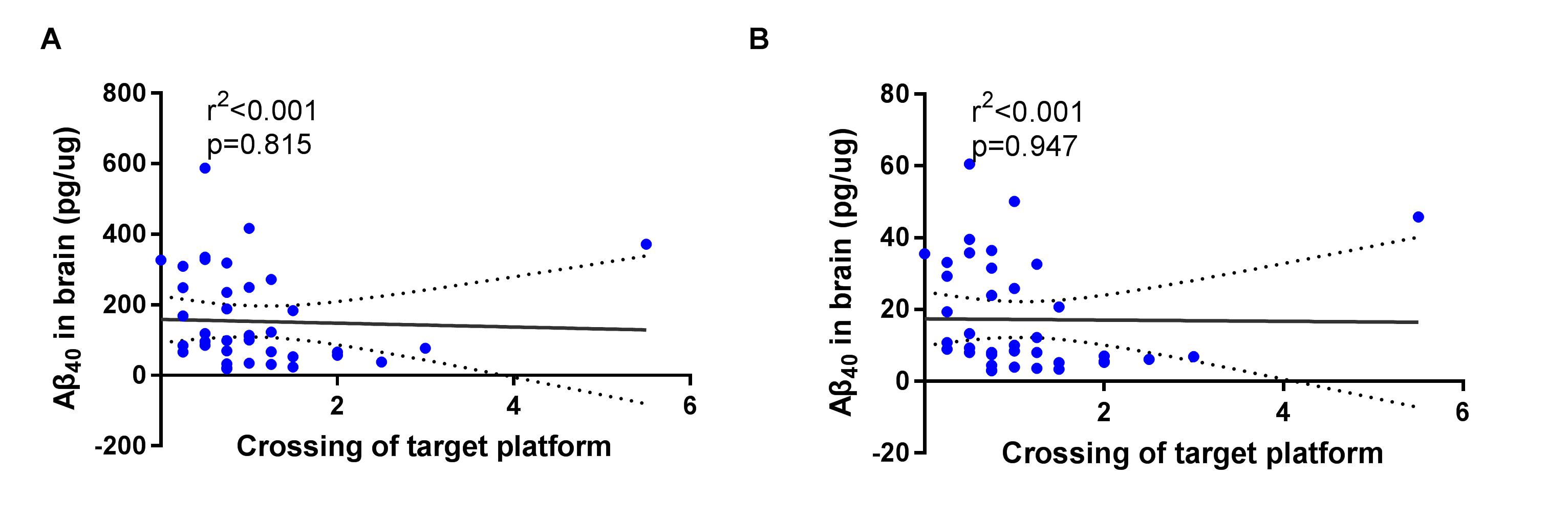


**Figure S2. Correlations between the times of target platform crossing and the concentration of Aβ_40_ (A) or Aβ_42_ (B) APP/PS1 mice.** Linear regression (n = 40 unless otherwise stated). r^2^ and p values from linear regression are represented in each panel.


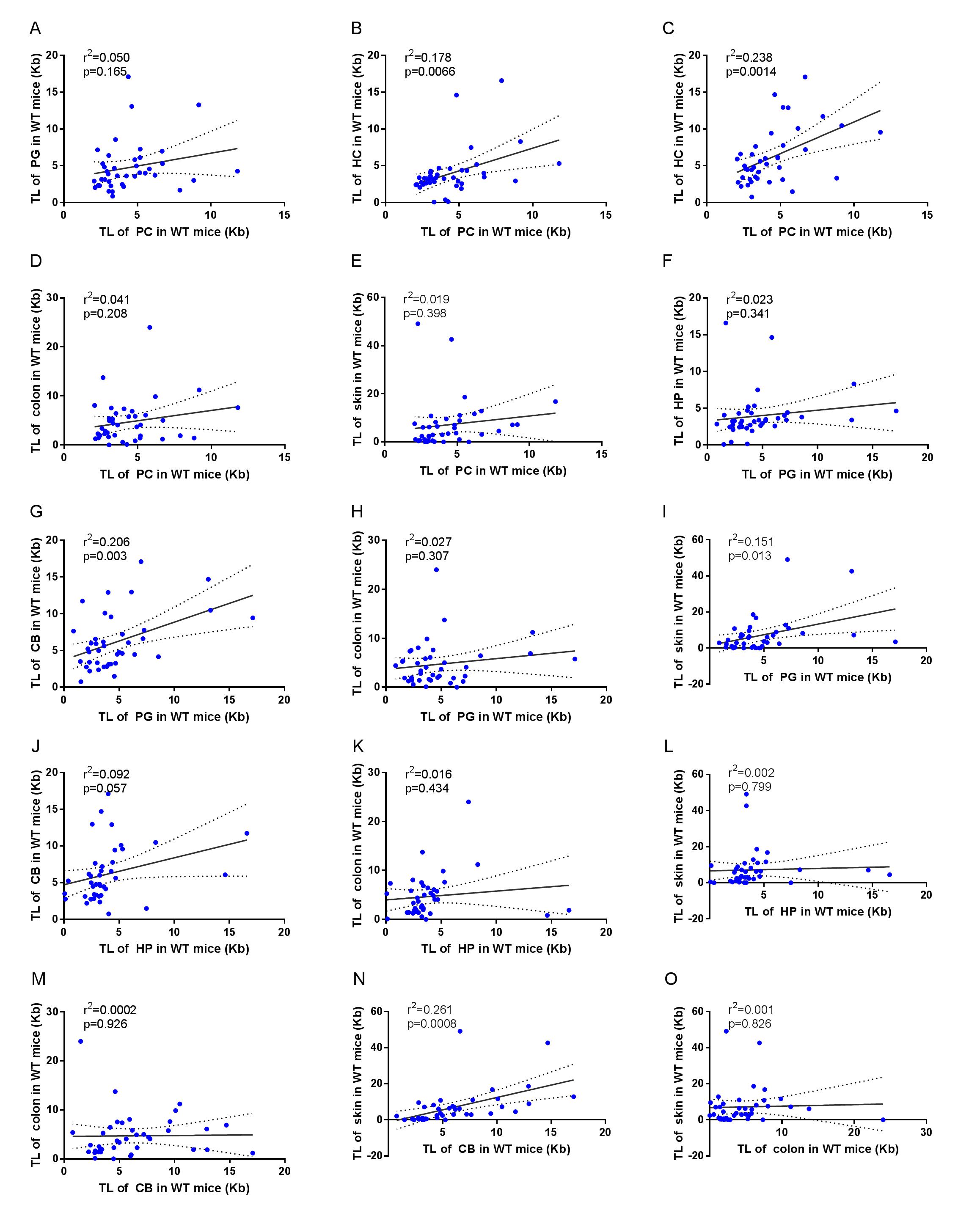


**Figure S3. Correlations of TL from different tissues in WT mice.** Linear regression (n = 40 unless otherwise stated). r^2^ and p values from linear regression are represented in each panel. Abbreviations: HC, hippocampus; PC, prefrontal cortex; CB, cerebellum; PG, pituitary gland.


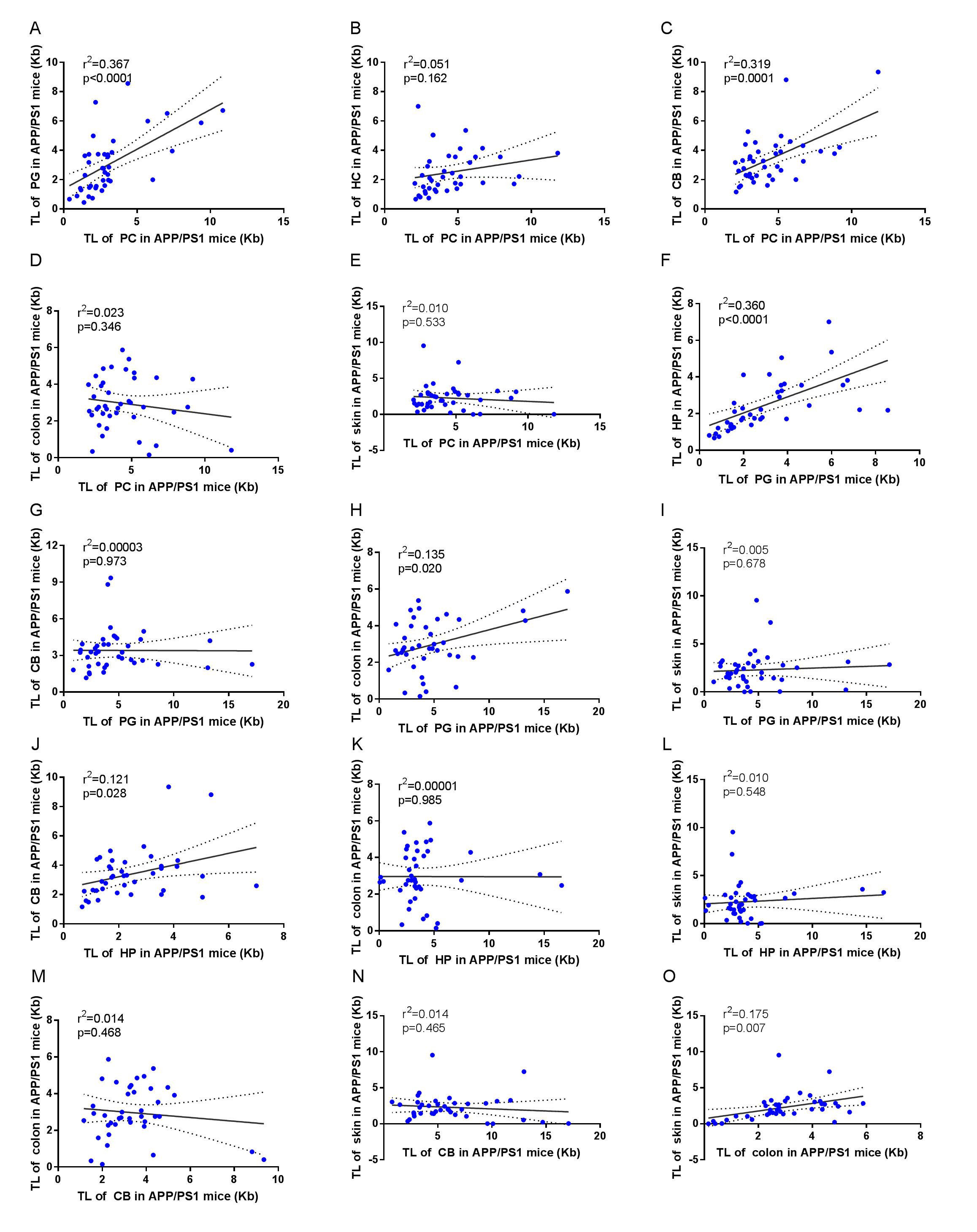


**Figure 4. Correlations of TL from different tissues in APP/PS1 mice.** Linear regression (n = 40 unless otherwise stated). r^2^ and p values from linear regression are represented in each panel. Abbreviations: HC, hippocampus; PC, prefrontal cortex; CB, cerebellum; PG, pituitary gland.


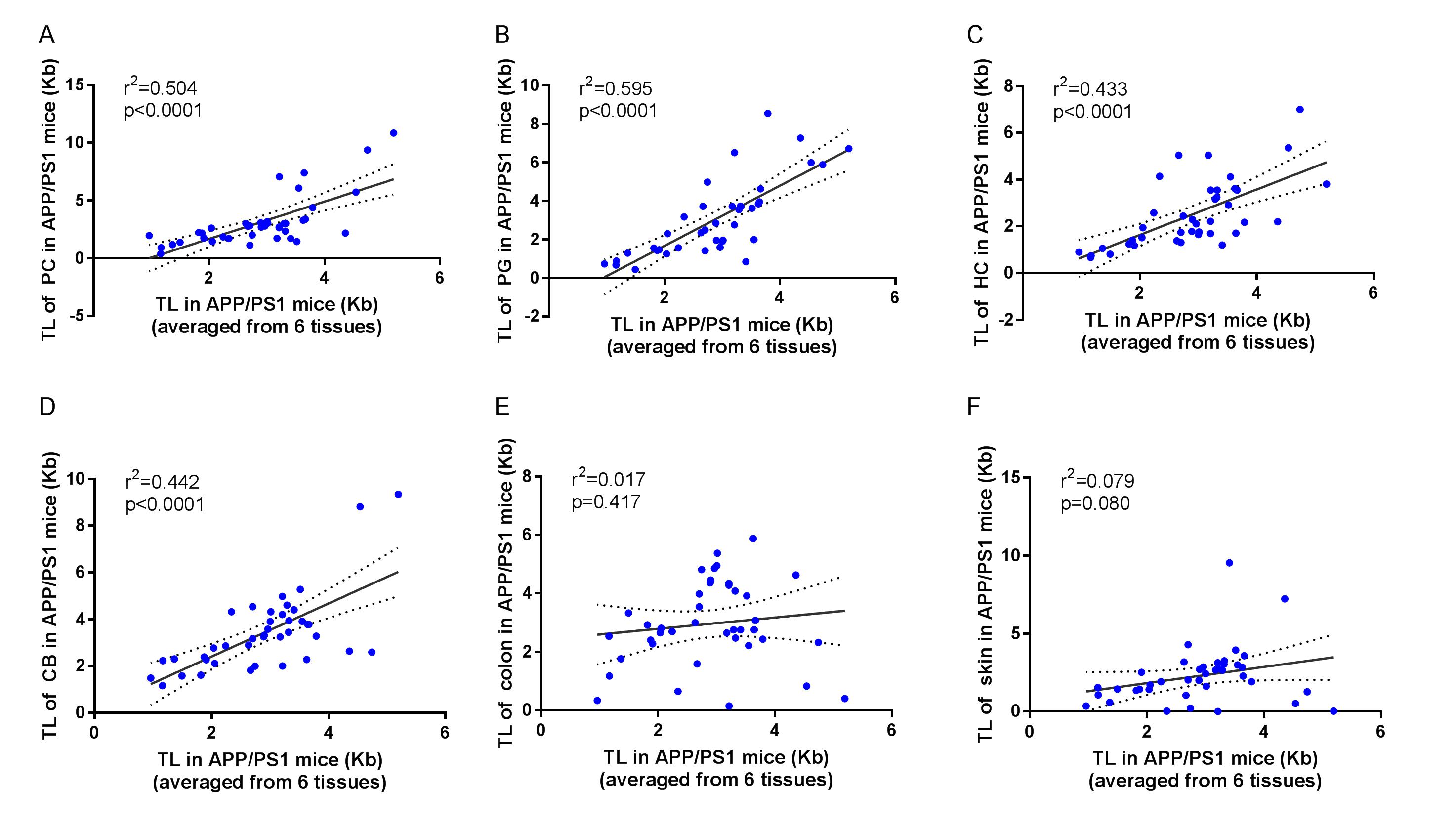


**Figure S5. Correlations between TL from different tissues with the mean TL in APP/PS1 mice.** Linear regression (n = 40 unless otherwise stated). r^2^ and p values from linear regression are represented in each panel. Abbreviations: HC, hippocampus; PC, prefrontal cortex; CB, cerebellum; PG, pituitary gland.


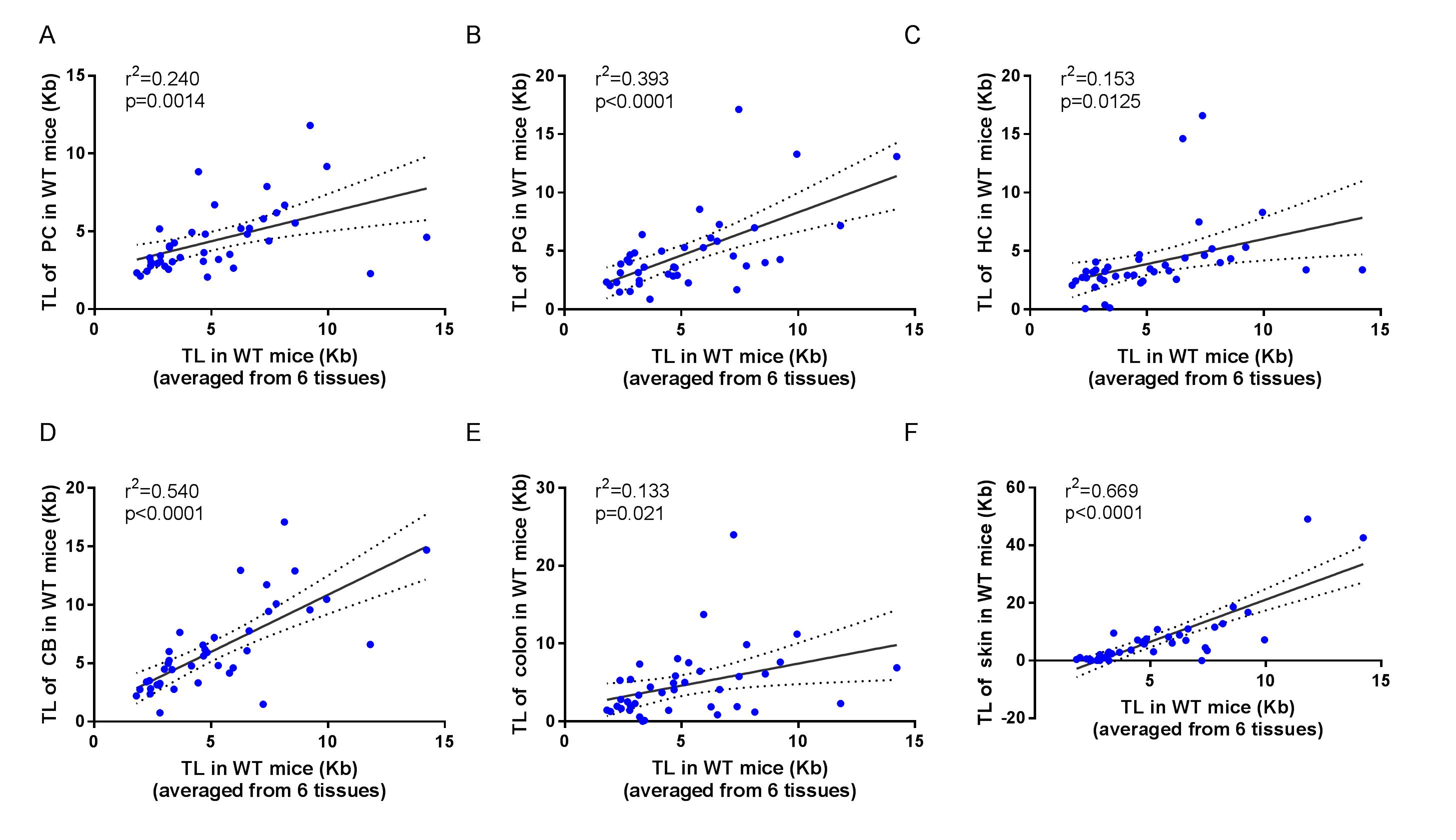


**Figure S6. Correlations between TL from different tissues with the mean TL in WT mice.** Linear regression (n = 40 unless otherwise stated). r^2^ and p values from linear regression are represented in each panel. Abbreviations: HC, hippocampus; PC, prefrontal cortex; CB, cerebellum; PG, pituitary gland.


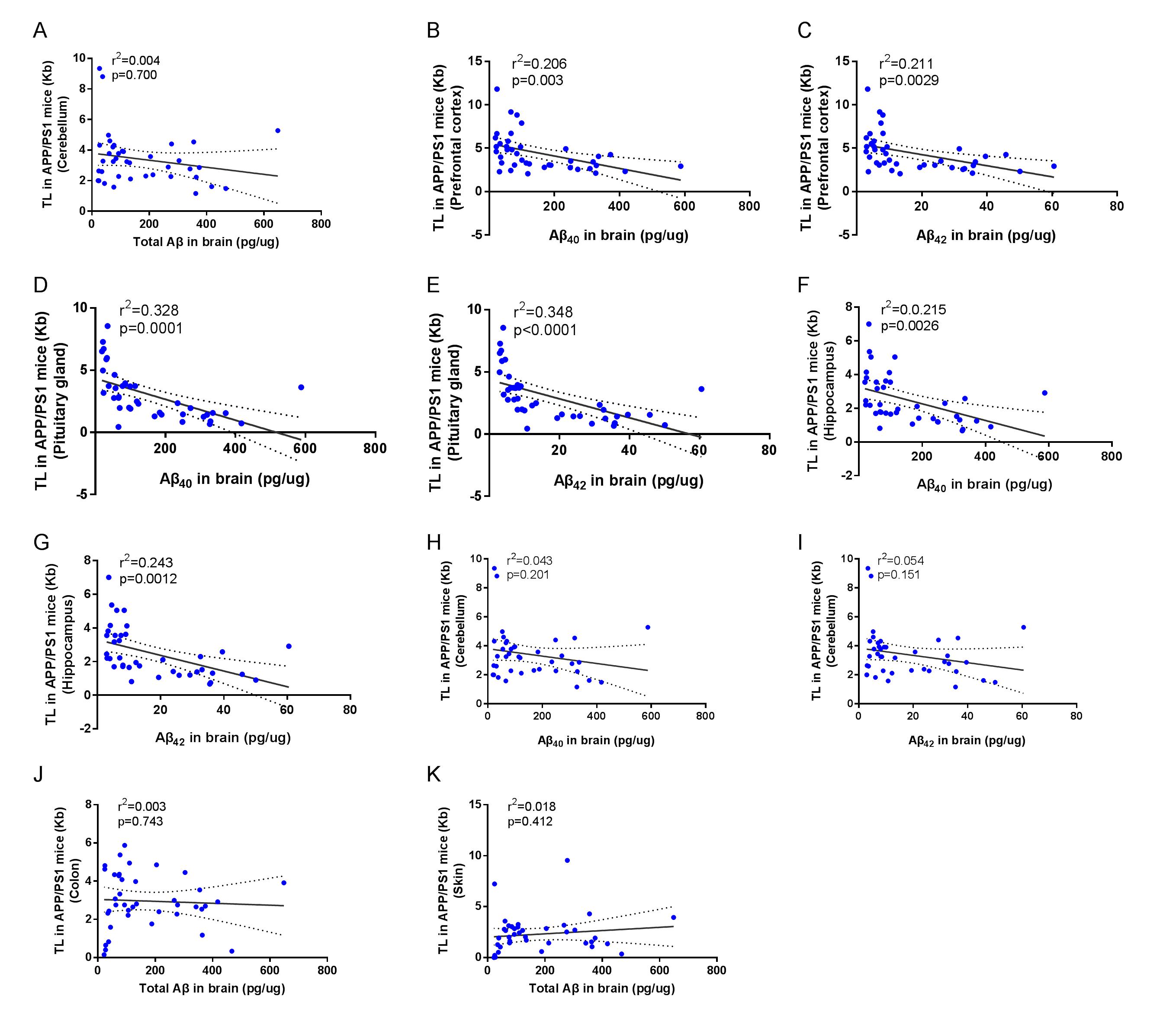


**Figure S7.** **Correlations between telomere length (TL) of specific tissue type with Aβ40, Aβ42 or total Aβ in APP/PS1 mice.** Linear regression (n = 40 unless otherwise stated). r^2^ and p values from linear regression are represented in each panel.


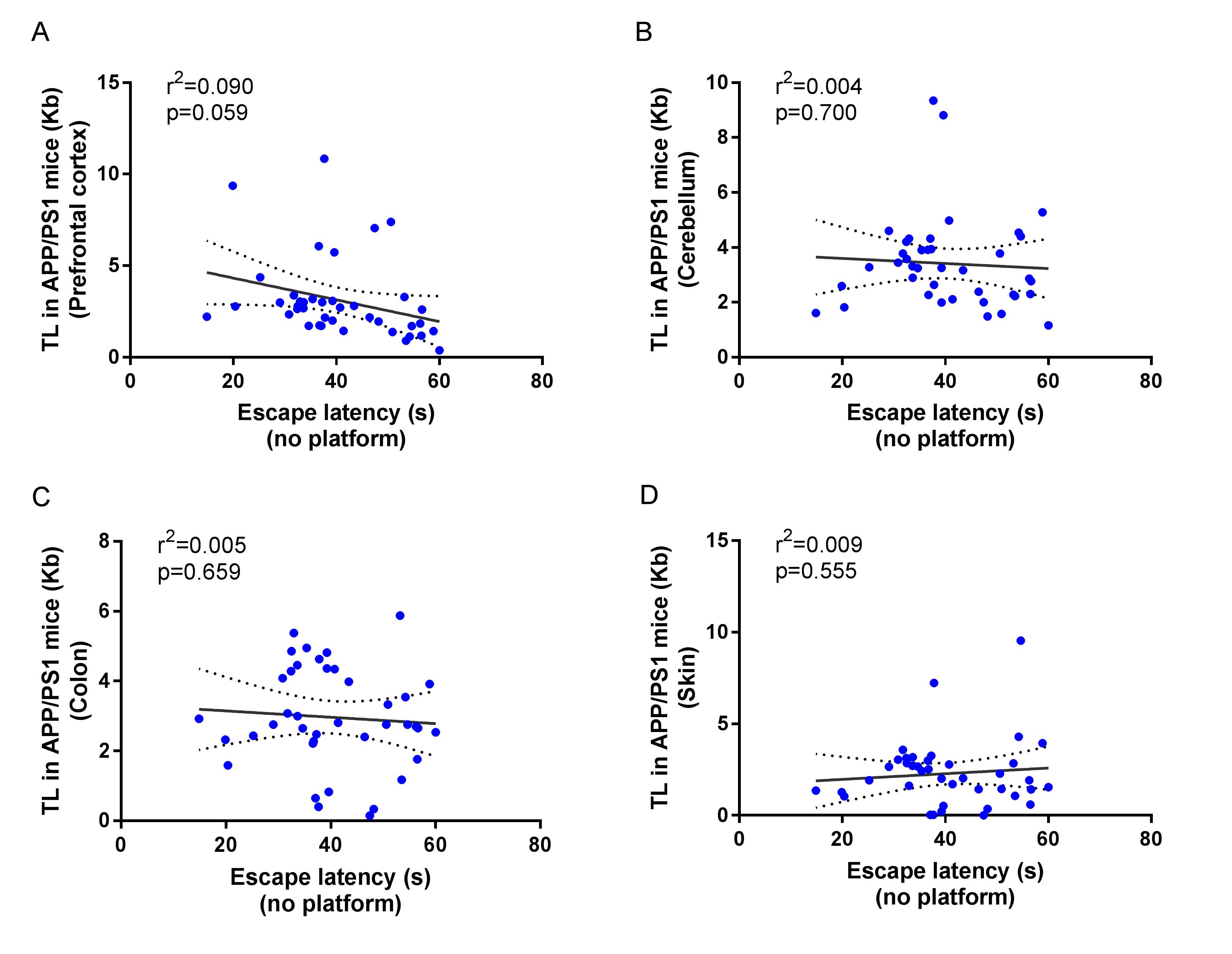


**Figure S8.** **Correlations between telomere length (TL) of specific tissue type with cognition performance in APP/PS1 mice.** Linear regression (n = 40 unless otherwise stated). r^2^ and p values from linear regression are represented in each panel.


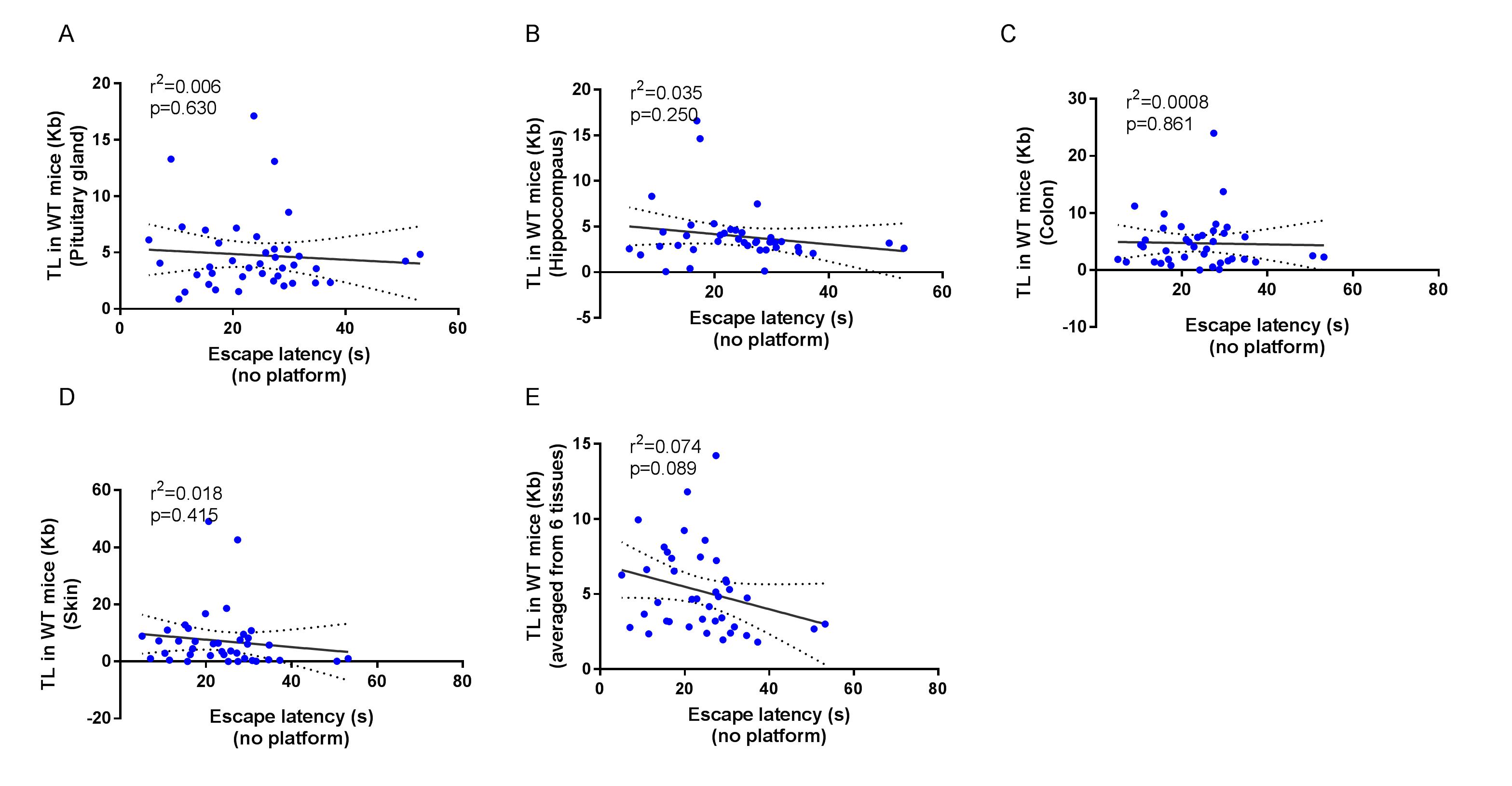


**Figure S9.** **Correlations between telomere length (TL) of specific tissue type or mena TL from 6 examined tissues with cognition performance in WT mice.** Linear regression (n = 40 unless otherwise stated). r^2^ and p values from linear regression are represented in each panel.


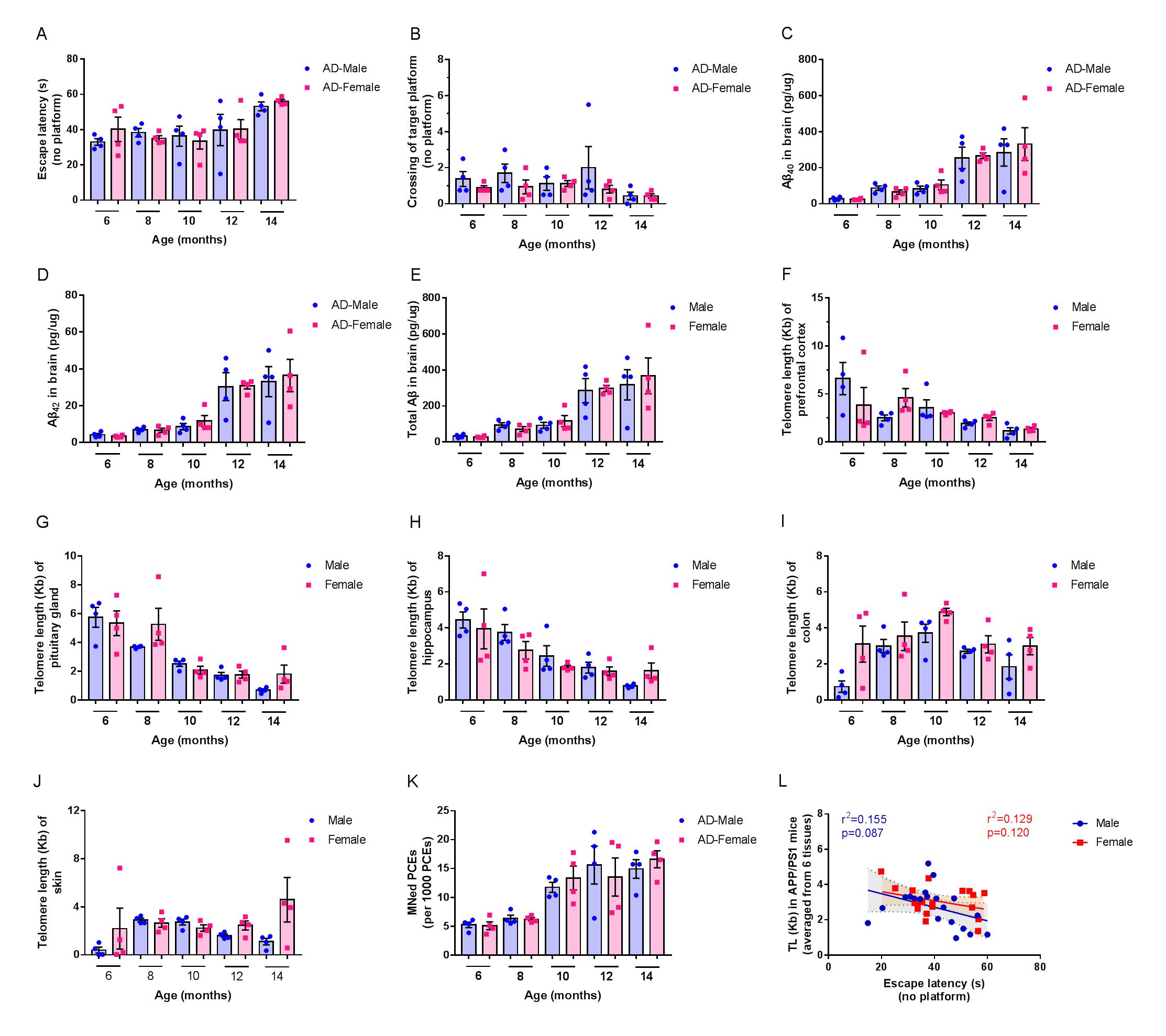


**Figure S10. Sex-related differences in APP/PS1 mice.** (A–K) The difference of cognitive performance (A and B), amyloidosis (C–E), telomere length (F–J) and micronuclei (K) frequency between females and males. (L) Correlation between mean telomere length (TL) from 6 examined tissues with cognition performance in females and males. Linear regression (n = 40 unless otherwise stated). All values in A–K are shown as mean ± SEM. Statistical analysis used in A-K a is two tailed Student’s t test. In L, r^2^ and p values from linear regression are represented.


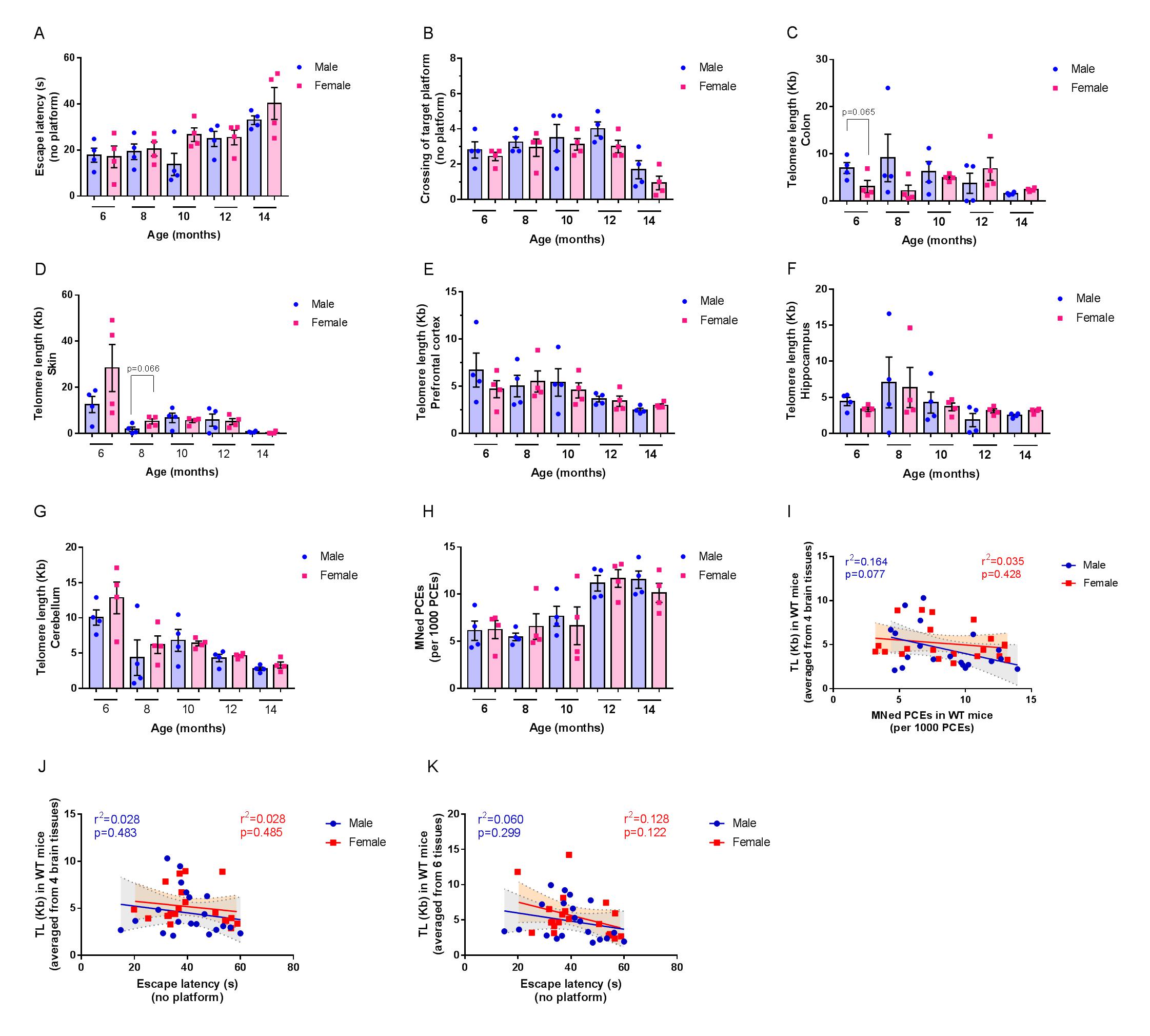


**Figure S11. Sex-related differences in WT mice.** (A-K) The difference of cognitive performance (A and B), telomere length (C-G) and micronuclei (H) frequency between females and males. (I) Correlation between mean telomere length (TL) from 4 brain tissues with micronuclei frequency in females and males. (J and K) Correlations between mean telomere length (TL) from 4 brain tissues (J) or from 6 examined tissues (K) with cognition performance in females and males. Linear regression (n = 40 unless otherwise stated). All values in A-H are shown as mean ± SEM. Statistical analysis used in A-H a is two tailed Student’s t test. In I-K, r^2^ and p values from linear regression are represented in each panel.
